# Fibronectin-based nanomechanical biosensors to map 3D surface strains in live cells and tissue

**DOI:** 10.1038/s41467-020-19659-z

**Published:** 2020-11-18

**Authors:** Daniel J. Shiwarski, Joshua W. Tashman, Alkiviadis Tsamis, Jaci M. Bliley, Malachi A. Blundon, Edgar Aranda-Michel, Quentin Jallerat, John M. Szymanski, Brooke M. McCartney, Adam W. Feinberg

**Affiliations:** 1 Department of Biomedical Engineering, Carnegie Mellon University, Pittsburgh, PA 15213 USA; 2 Department of Biology, Carnegie Mellon University, Pittsburgh, PA 15213 USA; 3 Department of Materials Science & Engineering, Carnegie Mellon University, Pittsburgh, PA 15213 USA

**Keywords:** Atomic force microscopy, Biosensors, Soft lithography, Biomedical engineering

## Abstract

Mechanical forces are integral to cellular migration, differentiation and tissue morphogenesis; however, it has proved challenging to directly measure strain at high spatial resolution with minimal perturbation in living sytems. Here, we fabricate, calibrate, and test a fibronectin (FN)-based nanomechanical biosensor (NMBS) that can be applied to the surface of cells and tissues to measure the magnitude, direction, and strain dynamics from subcellular to tissue length-scales. The NMBS is a fluorescently-labeled, ultra-thin FN lattice-mesh with spatial resolution tailored by adjusting the width and spacing of the lattice from 2–100 µm. Time-lapse 3D confocal imaging of the NMBS demonstrates 2D and 3D surface strain tracking during mechanical deformation of known materials and is validated with finite element modeling. Analysis of the NMBS applied to single cells, cell monolayers, and *Drosophila* ovarioles highlights the NMBS’s ability to dynamically track microscopic tensile and compressive strains across diverse biological systems where forces guide structure and function.

## Abstract (web summary)

The ability to measure strain in cells and tissues in vitro with minimal perturbation and at high spatial resolution has proven challenging. Here the authors develop a fluorescently-labelled fibronectin square lattice mesh that can be applied to the surface of cells and tissues to enable direct quantification and mapping of strain over time.

## Introduction

Cell-generated mechanical forces transmitted via cell-cell and cell-extracellular matrix (ECM) interactions are integral to a wide range of processes, from cell migration^1–6^ and contractility^7^ to tissue morphogenesis^8–14^ and regeneration^15–17^. Many diseases states are also characterized by impaired force generation, such as dilated cardiomyopathy^18^, or changes in ECM mechanical properties that, in turn, alter mechanosensitive gene expression, such as in fibrosis^19^. Yet in most cases the underlying mechanobiology is not well understood, in part because direct measurement of forces in living tissue during dynamic processes has proved difficult. Further, cells and tissues are spatially heterogeneous, with properties that can vary over the span of a few micrometers up to centimeters, and with forces generated on the cellular scale that combine to drive macroscale tissue formation^20,21^. A major challenge in the field is to directly map these cellular and tissue-level mechanical forces in 3D in both in vitro and in vivo systems. To do this, we need to develop a measurement tool that can span multiple length scales to enable force tracking, while minimally perturbing the biology of interest. However, forces do not need to be measured directly, and instead deformation tracking has been used widely to determine strain and calculate stress and related material and biomechanical properties.

There have been a number of important advances to measure strain in cells and tissues using computational, optical and combined methods. Finite element modeling and related computational techniques such as digital image correlation and finite element analysis (FEA) have been used to determine strain fields and estimate stresses based on known or approximated material properties^22–25^. Advanced optical techniques have also been developed including live cell imaging-based optical sensors (Förster resonance energy transfer, FRET)^26^, fluorescence based oil microdroplets^21^, 2D and 3D traction force microscopy^24,25,27^, birefringence^28^, and force inference^29^. Together, these methods have advanced the fields of biomechanics and mechanobiology, and provided insights into the role of biological forces in cell and tissue development and function. However, experimental limitations such as cell and tissue perturbation, limited field-of-view, sparse measurement density, and significant computational overhead, have spurred the continued development of newer approaches. There remains a need for a method that can combine (i) direct measurement of strain, (ii) high spatial and temporal resolution, (iii) tracking and mapping in both 2D and 3D, and (iv) minimal perturbation of the cell or tissue system.

Here we develop a fibronectin (FN) based nanomechanical biosensor (NMBS) that provides the capability to quantify the location, direction, and magnitude of strain on a 3D surface over time, from subcellular to tissue length scales. The NMBS is composed of a thin mesh (~10 nm thick) of fluorescently-labeled FN that can readily adhere to and integrate onto the surface of cells and tissues. The fluorescent NMBS mesh provides fiducial markers to track strain dynamically during developmental and physiological processes via live 3D fluorescence imaging. We engineer the NMBS utilizing an adaptation of our surface-initiated assembly (SIA) technique, which can be used with a range of ECM proteins (e.g., collagen type IV, laminin)^30^ and mesh micropattern designs depending upon experimental needs^31^. Prior knowledge of the tissue mechanical properties is not required, and spatial resolution of the NMBS is tuned by defining the width and spacing of the fibers that make up the mesh from 2 μm to 100 μm, enabling measurements from <1 μm to >1000 μm. For 3D image segmentation, tracking, and mapping of NMBS deformation over time as strain, we analyze confocal and multiphoton datasets using a custom open-source computational and visualization software package. Validation of the NMBS performance is achieved using materials of known material properties combined with FEA of deformation fields. Finally, we demonstrate the ability to directly quantify cell-generated 3D tensile and compressive mechanical strains at both cellular and multicellular resolution by applying the FN-based NMBS to the surface of single cells, cell monolayers, and developing tissues.

## Results

### Fabrication of fibronectin-based nanomechanical biosensors (NMBS)

The NMBS is fabricated using an adaptation of surface-initiated assembly (SIA), which is a technique to microcontact print ECM proteins onto a thermo-responsive poly(N-isopropylacrylamide) (PIPAAm) surface and then release the ECM proteins (e.g., FN, laminin, collagen type IV, and fibrinogen) as an assembled, insoluble network with defined geometry^31^. Our previous work has demonstrated that SIA can be used to engineer sheets of ECM proteins to shrink wrap cells in a defined ECM niche^32^, create a basement membrane to enhance endothelial cell growth^33^, and to micropattern ECM proteins on to microtopographies^30^. Here, instead of creating an ECM scaffold, we have repurposed the SIA approach to engineer the NMBS. Computer-aided design (CAD) software is used to define the geometry of the mesh, and in this initial work, we selected a square-lattice mesh because we can easily change the width and spacing of the fibers based on the experimental purpose.

**Fig. 1:**
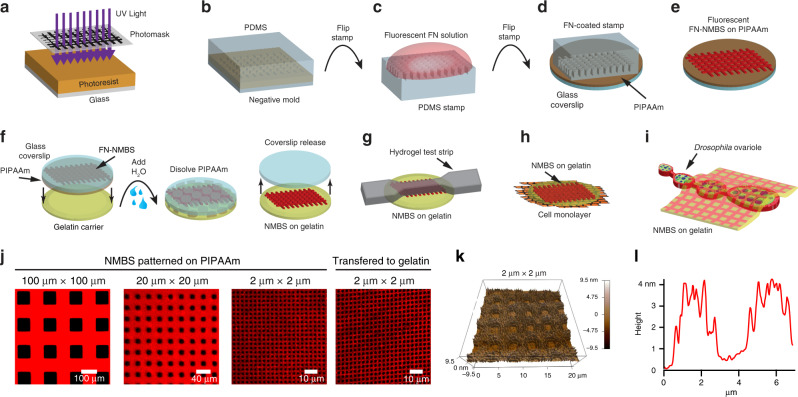
Microfabrication, experimental set-up, and analysis of the nanomechanical biosensor (NMBS). **a** Exposure of photoresist-coated glass wafer to ultraviolet (UV) light through a custom mesh photomask. **b** Casting of polydimethylsiloxane (PDMS) over topographically patterned photoresist-coated glass wafer. **c** PDMS stamp coated with fluorescently-labeled fibronectin (Alexa-555-FN, 50 µg/mL) solution. **d** FN-coated stamp is microcontact-printed onto a poly(N-isopropylacrylamide) (PIPAAm)-coated glass coverslip. **e** NMBS is patterned on the sacrificial substrate PIPAAm. **f** The NMBS-PIPAAm coverslip is placed NMBS-side down onto a gelatin type A carrier. Water is added to dissolve the PIPAAm and transfer the NMBS to the gelatin which releases the glass coverslip. **g**–**i** The NMBS is applied to dog bone hydrogel test strips (**g**), the top surface of cell monolayers (**h**), and the surface of *Drosophila* ovarioles (**i**) by placing the NMBS-gelatin carrier NMBS-side down on to the sample and raising the temperature to 37 °C to melt the gelatin and integrate the NMBS with the sample. **j** Alexa-647 fibronectin NMBS of varying resolution from large 100 µm × 100 µm to small 2 µm × 2 µm mesh sizes patterned onto PIPPAm and transferred onto gelatin. **k** Atomic force microscopy (AFM) height retrace image showing the topology of the patterned NMBS. **l** Line plot from the NMBS height retrace reveals an average thickness for the fibronectin NMBS of ~4 nm.

Briefly, the NMBS is designed in CAD, transferred to a transparency photomask, and used to photolithographically pattern a photoresist-coated glass wafer (Fig. 1a). A polydimethylsiloxane (PDMS) elastomer stamp is formed by casting on the patterned wafer (Fig. 1b) and is then inked with fluorescently-labeled FN (Fig. 1c). The PDMS stamp is then used to microcontact print the FN onto a PIPAAm-coated glass coverslip (Fig. 1d) and then removed to leave the NMBS on the PIPAAm surface (Fig. 1e). To conform to the topology of cells and tissues, a gelatin carrier film is brought in contact with the NMBS-patterned PIPAAm-coated coverslip and then room temperature water is added to dissolve the PIPAAm and transfer the NMBS to the gelatin (Fig. 1f). The NMBS is then applied to various sample types, including tensile test strips (Fig. 1g), cell monolayers (Fig. 1h), and developing tissues (Fig. 1i). In each case the NMBS is transferred by incubation at 37 °C for 5 min to melt the gelatin, integrating the NMBS onto the desired surface. The NMBS can be tailored with a wide range of dimensions, but we found that line width and spacing of 2 µm × 2 µm, 20 µm × 20 µm, 10 µm × 100 µm, and 100 µm × 100 µm could measure strain from the subcellular to multicellular length scales and be transferred to the gelatin carrier with high fidelity (Fig. 1j). Finally, the thickness of the dry NMBS was ~4 nm by atomic force microscopy (Fig. 1k, l), verifying nanoscale fabrication of an ultra-thin mesh that should minimally perturb the surface it is attached to.

### Validation of the NMBS to track strain

**Fig. 2:**
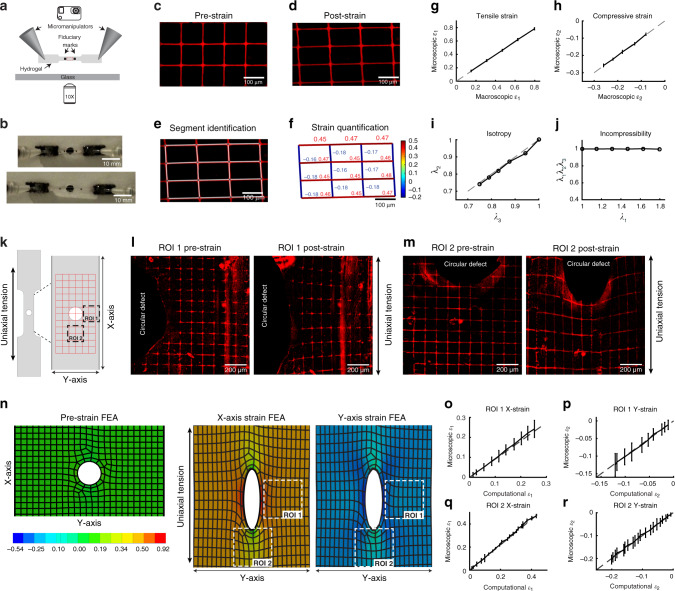
Uniaxial tensile testing and finite element analysis of the nanomechanical biosensor (NMBS) on a polydimethylsiloxane (PDMS) test strip. **a** A PDMS dog bone test strip with applied NMBS is mounted on micromanipulators attached to a wide-field fluorescence microscope to view the NMBS (100 µm × 100 µm × 10 µm) from underneath while tracking fiducial marks on the top surface during uniaxial tensile testing. **b** Example images of the PDMS test strip in the pre-strain and post-strain states. **c** Representative fluorescence image of pre-strained NMBS prior to tensile testing. **d** Representative fluorescence image of deformed NMBS following tensile testing. **e** Image segmentation and analysis is performed to identify rectangular mesh NMBS nodes and segments are then overlaid onto the deformed image. **f** Segment lengths are converted into tensile (positive) and compressive (negative) mechanical strains with respect to their pre-strain reference length. A color map reveals locations of tensile (red) and compressive (blue) mechanical strains. **g** The microscopic tensile strain ε_1_ of the NMBS strongly correlates with the macroscopic tensile strain ε_1_ of PDMS (mean ± S.D.; *n* = 12 segments over 1 experiment). **h** The microscopic compressive strain ε_2_ of the NMBS strongly correlates with the macroscopic compressive strain ε_2_ of PDMS (mean ± S.D.; *n* = 12 segments over 1 experiment). **i** There is a strong correlation between the stretch ratios λ_2_ and λ_3_ confirming that PDMS is an isotropic material. **j** The product of the stretch ratios λ_1_λ_2_λ_3_ is equal to 1 confirming that PDMS is incompressible. **k** A circular defect in the PDMS test strip was introduced to track heterogeneous strain fields with the NMBS during uniaxial tensile testing. **l** Representative fluorescence images from ROI 1 (region adjacent to the circular defect and antiparallel to the uniaxial tension) of the NMBS pre and post tensioning. **m** Representative fluorescence images from ROI 2 (region adjacent to the circular defect and parallel to the uniaxial tension) of the NMBS pre and post tensioning. **n** Finite element analysis of a PDMS test strip with a circular defect in the pre-strain, X-strain, and Y-strain states following 40% uniaxial strain (blue = compression; green = no strain; red = tension). **o** Quantitative analysis of ROI 1 reveals a strong correlation between the microscopic X-strain ε_1_ of the NMBS with the computational tensile X-strain ε_1_ (mean ± S.D.; *n* = 28 segments over 1 experiment). **p** Additionally, there is a strong correlation between the microscopic Y-strain ε_2_ of the NMBS with the computational compressive Y-strain ε_2_ (mean ± S.D.; *n* = 30 segments over 1 experiment). **q** Quantitative analysis of ROI 2 reveals a strong correlation between the microscopic X-strain ε_1_ of the NMBS with the computational tensile X-strain ε_1_ (mean ± S.D.; *n* = 8 segments over 1 experiment). **r** Additionally, there is a strong correlation between the microscopic Y-strain ε_2_ of the NMBS with the computational compressive Y-strain ε_2_ (mean ± S.D.; *n* = 9 segments over 1 experiment).

To validate and benchmark the NMBS as a microscopic strain sensor, we performed controlled mechanical testing while visualizing microscopic deformations through fluorescence microscopy. First, a PDMS “dog bone” shaped tensile test strip was designed and molded (Supplementary Fig. 1) and NMBS with 10 µm wide lines and 100 µm spacing was transferred across the gauge length (Fig. 1g). This test strip was then mounted in a custom-designed uniaxial testing system that enables simultaneous microscopic imaging of the NMBS and macroscopic imaging of the PDMS (Fig. 2a). Elongation of the PDMS test strip was measured by an increased distance between the fiducial marks (small black dots on the PDMS in the gauge length, Fig. 2b). Fluorescence images of the NMBS pre-strain (Fig. 2c) and following 45% uniaxial strain (Fig. 2d) show the microscopic deformations tracked by the NMBS mesh. The initial observation state, *l*_0_, is considered as the zero-strain reference configuration. Subsequent deformation of the NMBS, *l*, relative to the initial reference state, *l*_0_, enables calculation of engineering strain as $\varepsilon = \frac{l}{{l_0}} - 1$ within each NMBS segment. As a single NMBS filament segment between nodes of the mesh approximates a 1-dimensional strain sensor, the resultant strain obtained corresponds to the strain along each segment. The combination of all filaments within the NMBS, which are in multiple orientations, provides information about the 3-dimensional strain state. Time-lapse fluorescence imaging of the NMBS provides tracking of both tensile and compressive strain at the microscopic scale based on mesh deformation. Quantification of strain is performed using custom MATLAB software by converting the NMBS fluorescence image into a binary image, identifying intersection nodes, connecting the nodes to form a rectangular mesh, and overlaying it onto the original image (Fig. 2e). Node-to-node distances are measured as segment lengths for calculation of engineering strain to demonstrate tensile (positive) and compressive (negative) strains with respect to their undeformed reference length (Fig. 2f). The segments are further color coded according to their strain value to obtain a global map of the tensile and compressive strains (Fig. 2f).

To validate the accuracy of the NMBS strain mapping during uniaxial tensile testing, we correlated the average tensile and compressive strain observed via fluorescence microscopy to the measured macroscopic distances between fiducial marks on the surface of the PDMS test strip. We also measured the overall width and thickness of the test strip throughout tensile testing. The mean and standard deviation of the microscopic tensile ε_1_ (parallel to uniaxial testing) and compressive ε_2_ (perpendicular to uniaxial testing) strains of NMBS were plotted against the macroscopic tensile ε_1_ (Fig. 2g) and compressive ε_2_ (Fig. 2h) strain calculated from the fiducial marks and strip dimensions. In each case, the microscopic strain measurement was highly correlated with the macroscopic strain measurement (Pearson correlation coefficient of 0.999), establishing that the NMBS accurately tracks strain. We can also use the stretch ratio, defined as $\left( {\lambda = \frac{l}{{l_0}}} \right)$, to investigate material properties of the PDMS. The stretch ratios λ_2_ and λ_3_ were highly correlated (Pearson correlation coefficient of 0.998, slope = 1) (Fig. 2i), and the product of the stretch ratios λ_1_λ_2_λ_3_ was equal to 1 at all steps of the uniaxial stretching (Fig. 2j), indicating that PDMS behaves as an isotropic incompressible material in agreement with the literature^34^. To evaluate the reproducibility, we performed uniaxial tensile testing with 45% strain loading-unloading cycles at 3% increments. The microscopic tensile ε_1_ and compressive ε_2_ strain measurements remained highly correlated with the macroscopic tensile ε_1_ and compressive ε_2_ strain measurements (Pearson correlation coefficient of 0.999) (Supplementary Fig. 2).

For large uniform deformation, macroscopic fiducial marks are sufficient for tracking strain during uniaxial tensile testing, but they do not provide the ability to measure heterogeneous strains at high resolution. To evaluate the NMBS for this purpose, we tracked microscopic strain fields surrounding a circular defect within a PDMS test strip during tensile testing and focused on two regions of interest (ROI 1, ROI 2) (Fig. 2k). Following 40% uniaxial strain, ROI 1 (lateral to the defect) showed X-axis tensile strain ε_1_ and Y-axis compressive strain ε_2_ increased next to the defect (Fig. 2l). Similarly, ROI 2 (longitudinal to the defect) showed X-axis tensile strain ε_1_ and Y-axis compressive strain ε_2_ decreased next to the defect (Fig. 2m). We used FEA to model uniaxial testing of the PDMS with a 0.4 mm circular defect, taking advantage of the PDMS having well characterized mechanical properties and behaving as an ideal elastomer. To visually compare the deformations tracked by the NMBS to the simulated results, we chose to use element sizes for FEA of the PDMS that matched the size and shape of the NMBS. Simulations of the uniaxial tensile testing confirmed our experimental results showing increased X-axis tensile strain ε_1_ and Y-axis compressive strain ε_2_ near the defect in ROI 1, and decreased X-axis tensile strain ε_1_ and Y-axis compressive strain ε_2_ near the defect in ROI 2 (Fig. 2n). Importantly, there was high correlation (Pearson correlation coefficient of 0.999) between the microscopic strain observed using the NMBS and FEA-simulated strain within the X-axis and Y-axis for both ROI 1 and ROI 2 (Fig. 2o–r). Taken together, these data demonstrate the ability of the NMBS to accurately track and quantify both homogenous and heterogeneous strains at the microscale.

As a final validation step, we used the NMBS to track strain of a fibrin tensile test strip as a model of a biologically-derived compressible hydrogel. Fluorescent images of the NMBS in its reference state (Supplementary Fig. 3a) and following 50% uniaxial strain (Supplementary Fig. 3b) demonstrated the ability of the NMBS to adhere to and track microscopic strain. Following 50% uniaxial strain of the fibrin test strip, quantification of individual segment strain and mapping revealed an average tensile strain of 0.4938 ± 0.0238 in the stretch direction and an average compressive strain of 0.5188 ± 0.0403 in the orthogonal direction (Supplementary Fig. 3c, d). Further, the NMBS microscopic strain measurement was highly correlated with the macroscopic strain measurements (Pearson correlation coefficient of 0.999) (Supplementary Fig. 3e, f). Unlike PDMS, fibrin test strips stretch ratios λ_2_ and λ_3_ were not equivalent (Supplementary Fig. 3g; slope = 1.42), and the product of the macroscopic stretch ratios λ_1_λ_2_λ_3_ was less than 1 at all steps of the uniaxial stretching (Fig. 2h) indicating that fibrin behaves as a compressible material^35^. This demonstrates that the NMBS can accurately track heterogeneous microscopic strain on samples with different material properties.

### Mapping subcellular, cellular and multicellular strain with the NMBS

**Fig. 3:**
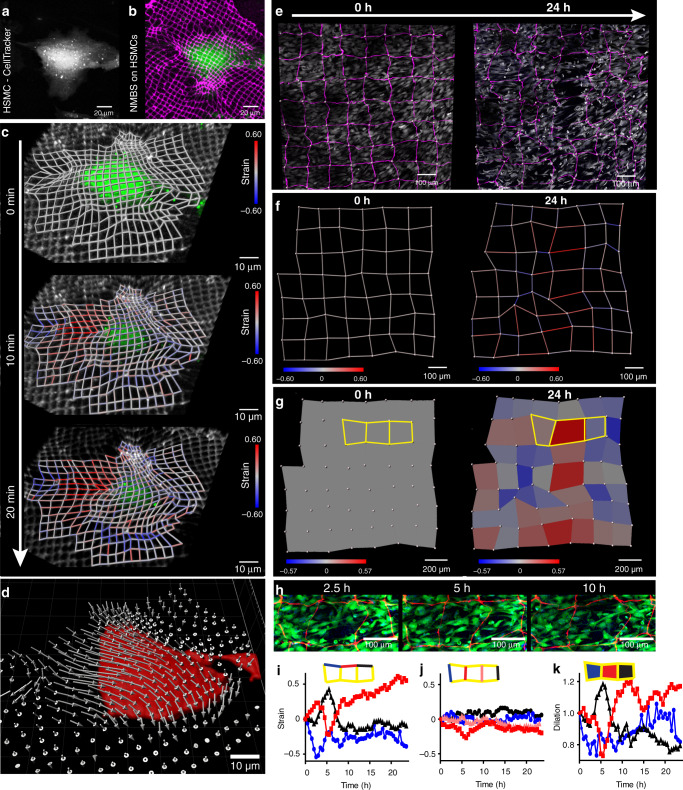
3D cellular strain mapping with the NMBS. **a** Human skeletal muscle cells (HSMC) visualized with CellTracker-488. **b** Alexa-647 fibronectin NMBS (2 µm × 2 µm × 2 µm, magenta) applied to the HSMC (green). **c** Mapping of the NMBS strain over time reveals local regions of tension and compression following cell migration and contraction. **d** 3D displacement vector map (gray arrows) showing the magnitude and direction of displacement for all NMBS mesh nodes as the cell compresses (Red surface) over 20 min. **e** Alexa-555 fibronectin NMBS (100 µm × 100 µm × 10 µm, magenta) applied to C2C12 cells visualized with CellTracker-488 (gray). Images were acquired every 30 min for 24 h to track strain following transition to C2C12 differentiation media. **f** Strain mapping of the NMBS (red = tension, blue = compression) following transition to differentiation media reveals localized regions of tension and compression. **g** Area dilation was calculated for each section of the NMBS over time and shows spatial regions of expansion and contraction. **h** Regions of interest extracted showing cellular elongation events at 2.5, 5, and 10 h following transition to differentiation media (NMBS, red; C2C12 cells, green). **i**, **j** Quantification of segments from the region of interest in “g” during the C2C12 differentiation process shows region-specific strain rates during the 24-h time course, with the largest magnitude strains occurring in the x-direction. **k** Time-dependent mapping of regional dilation during the C2C12 differentiation process for ROI analyzed in (**i**, **j**).

To apply the NMBS to biological systems we started at the single cell level. Traction force measurements have become a widely used methodology, but measurements are largely limited to forces applied to the basal cell-substrate interface, and the only way to get apical traction forces is to perform embedded 3D traction force microscopy^25,27,36–38^. The NMBS can be applied to the free apical surface of the cell to provide quantitative information on membrane deformation. To demonstrate this, the NMBS was applied to the apical surface of human skeletal muscle cells (HSMCs) grown in culture, using the 2 µm × 2 µm mesh to provide subcellular resolution. HSMCs were labeled with CellTracker™ Green for visualization (Fig. 3a) and the fluorescently-labeled NMBS conformed to and adhered to the cell surface (Fig. 3b). A custom image analysis pipeline was created using Imaris image analysis and MATLAB software to extract the 3D surface strain information from the NMBS deformation over time (Supplementary Fig. 4a–i). As a HSMC migrates over a 20 min period, tensile strain increases at the trailing edge and compressive strain increases at the leading edge (Fig. 3c and Supplementary Movie 1). Tracking and visualization of displacement vectors for each node within the NMBS reveal the magnitude and direction of movement for the apical membrane surface in 3D (Fig. 3d). Further analysis was performed to determine subcellular strain during migration. Nuclear, cytoplasmic, and peripheral membrane regions of the NMBS were defined to evaluate both the horizontal (X-axis) and vertical (Y-axis) strain components (Supplementary Fig. 4j) as the HSMC migrated during the imaging timecourse (Supplementary Fig. 4k). Quantification of the X-axis strain revealed an increase in compression for the nuclear region and in increase in variability going from the cytoplasmic to peripheral membrane regions (Supplementary Fig. 4l). Quantification of the Y-axis strain showed minimal change within the nuclear region and increased but similar variability within the cytoplasmic and peripheral membrane regions (Supplementary Fig. 4m). We also evaluated tensile strain at the trailing edge (ROI 1) and the region of compressive strain at the leading edge (ROI 2) and revealed average peak tensile strains of 110% ± 22% and compressive strains of 39% ± 9% along the membrane, as compared to an average strain of 3.4% ± 10% within an NMBS region adjacent to the cell (Supplementary Fig. 4n). Utilizing the NMBS to track cellular strain thus allows for the creation of an apical surface strain map with subcellular resolution.

To expand the technology to multicellular measurements we used the NMBS to track strain in C2C12 mouse myoblast monolayers, with the goal to study myoblast migration and fusion of single cells into multinucleated myotubes. The NMBS (10 µm wide fibers spaced at 100 µm) was applied to a confluent C2C12 monolayer labeled with CellTracker™ Green, and the media was changed to differentiation media. Over 24 h of live imaging, the C2C12 cells were observed to undergo coordinated and regionally localized migration into areas of high and low cell density, highlighted by the deformations within the NMBS (Fig. 3e and Supplementary Movie 2). Strain between nodes of the mesh quantified and mapped from the NMBS deformations showed large tensile and compressive strains correlating with the regions of low and high cell density, respectively (Fig. 3f). Analysis of dilation for the area within each square of the NMBS mesh produced a field map of the expansion and contraction (Fig. 3g). To look at the correlation between strain and the dynamics of cell migration and elongation, we further evaluated a region of interest that exhibited high tensile strain, large dilation, and oscillations over time (Fig. 3h and Supplementary Movie 3). Quantification revealed segment-specific periodic changes in the X-strain (Fig. 3i), with minimal change in the Y-strain (Fig. 3j). Dilation analysis of this region was correlated with the X-strain data (Pearson Correlation 0.806 ± 0.094), but not the Y-strain data (Pearson Correlation −0.206 ± 0.231) suggesting that the regional expansion and contraction observed during the C2C12 migration and elongation is driven by anisotropic, X-axis biased tension and compression (Fig. 3k). To further confirm that cell morphological changes were driving the NMBS deformation we quantified the cell motility and tracked trajectories over time. Looking at a subsection of the NMBS we demonstrate that the underlying cell motility and morphological changes parallel the strain deformations and regional changes in dilation measured via the NMBS (Supplementary Fig. 5a, b).

Our initial dilation analysis used the NMBS node locations and connecting segments to construct bounding boxes (Supplementary Fig. 5c), but for large segment lengths (>20 µm) significant deformations can occur within the segment itself. To quantify these changes, we can utilize the fluorescence confocal images of the NMBS to obtain a 2D projection image of the change in dilation over time. Using this approach, we observed a similar overall dilation field map over 12 h of C2C12 migration in response to differentiation media (Supplementary Fig. 5d) compared to the previous node to node analysis (Supplementary Fig. 5c); however, some regional variation can be observed where there was large deformation occurring within a segment (Supplementary Fig. 5d). A representation of the full time series of NMBS deformation is shown in Supplementary Fig. 5e. Together, these data demonstrate that the NMBS is an accurate fiducial marker for strain and area dilation. Future work will focus on incorporating more sophisticated point cloud-based analysis into our software package to enable true 3D surface area measurements without requiring 2D approximations.

### Dynamic strain measurement during cardiomyocyte contractions

**Fig. 4:**
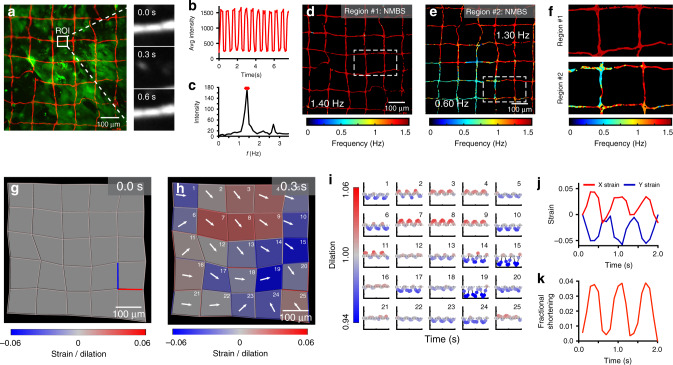
Beat frequency analysis and strain mapping during cardiomyocyte contractions. **a** Fluorescence image of representative Alexa-555 fibronectin NMBS (Red, 100 µm × 100 µm × 10 µm) applied to iPSC-derived cardiomyocyte monolayer loaded with Fluo-4 calcium indicator dye (Green). White box indicates ROI for NMBS motion analysis. Motion analysis of ROI displays NMBS segment moving in and out of the ROI over time. **b** Quantification of the average fluorescence intensity from the ROI through time. **c** Example power spectral density plot from the Fourier transformed data of a NMBS ROI segment showing the intensity at each frequency with a dominant frequency of 1.4 Hz. **d** A synthetic image is constructed containing the frequency information of every pixel within the NMBS to visualize variability of contractile frequencies for cardiomyocyte monolayers. A dominant uniform frequency of 1.4 Hz is observed. **e** Example region #2 of contractile cardiomyocytes demonstrating heterogeneous beat frequencies of 1.3 and 0.6 Hz. **f** Magnified images of region #1 and #2 demonstrating differences in heterogeneity between fields. **g**, **h** Time-series showing regional dilation and NMBS segment strain throughout a contraction cycle of diastole and systole. Displacement vectors (white arrows) for each square region defined by the NMBS show the contractile direction between peak systole and diastole. **i** Analysis of dilation over a 2 s time interval defined by the NMBS meshed regions (red = tension, blue = compression). **j** Quantitative analysis of example X (red line in **g**) and Y (blue line in **g**) strain from NMBS segments shows the tensile and compressive strain rate over time for cardiomyocytes with a beat frequency of 1.4 Hz, maximum tension of 4% and compression of 6%. **k** Fractional shortening is calculated from a regional dilation analysis of ROIs 13, 14, 18, and 19 demonstrating a peak fractional shortening of 3.8% ± 0.06 between diastole and systole (mean ± S.D.; *n* = 3 cycles over 1 experiment).

In many biological systems, cells and tissues undergo dynamic movements that drive developmental and physiological processes. This is especially true for cardiac tissue, where cardiomyocytes (CMs) display coordinated contraction and electromechanical coupling into a syncytium^39^. For CMs derived from human embryonic stem cells (hESCs), coordinated contraction and shortening are important markers of maturation and disease phenotype^40,41^. Heterogeneity following differentiation is also a critical quality metric, but difficult to measure in 2D culture settings^41^. Here we assessed hESC-CM function using the NMBS to measure beat frequency and contraction induced strain, combined with fluorescent imaging of calcium signaling (Fig. 4a and Supplementary Movie 4). Motion of the NMBS was used to extract CM beat frequency from each pixel region of the NMBS by measuring changes in average fluorescence intensity over time (Fig. 4a, b and Supplementary Movie 5). A fast Fourier transform (FFT) analysis was performed to extract the dominant frequency from the fluctuations in average fluorescence intensity over time (Fig. 4c). Using these values an image can be reconstructed as a region-specific CM beat frequency mapped to each pixel of the NMBS in the field of view (Fig. 4d). To validate this approach, the beat frequency of spontaneously contracting CMs calculated from NMBS motion was compared to the calcium signaling and found to be the same (Supplementary Fig. 6a). Similar results were found when paced with either 1 Hz or 2 Hz field stimulation. The NMBS and calcium wave frequency data were identical, and both matched the stimulation rate (Supplementary Fig. 6b). Mapping the beat frequency on to the NMBS showed that it was uniform across the field of view at 2 Hz stimulation (Supplementary Fig. 6c).

Next, hESC-CM cultures were evaluated for beat frequency heterogeneity using the NMBS mapping. The typical field of view revealed homogeneous contractions (Fig. 4d), which is indicative of good cell-cell coupling between CMs and the formation of a continuous syncytium. However, there were also fields of view that had heterogeneity (Fig. 4e) with variable beat frequencies ranging from approximately 0.5 Hz to 1.5 Hz (Supplementary Movie 6). Transitions between beat frequency rates appeared to occur at sharp boarders within continuous cell monolayers and were seen to differ between fields of view within a single coverslip (Fig. 4f and Supplementary Movie 7). To obtain enhanced resolution for beat frequency analysis, we also used an NMBS mesh with a smaller gird size consisting of 10 µm wide fibers separated by 20 µm and compared this to the calcium signaling. The increased resolution provided by the denser NMBS mesh pattern, together with the calcium signaling, revealed that the heterogeneity in beat frequency was due in part to other cell types that were not hESC-CMs in the culture. Specifically, regions that had beat frequencies from 0 to 0.5 Hz did not show calcium signaling (Supplementary Fig. 6d), indicating that these were not hESC-CMs. To support this, fields of view that had uniform beat frequency determined by the NMBS also showed uniform calcium signaling. Differentiation of hESC-CMs is ~90% efficient, so non-contractile cells are expected. The advantage of the NMBS over calcium imaging, however, is that we can assess beat frequency and heterogeneity without needing to use an intracellular fluorescent stain that has the potential to cause phototoxicity.

In addition to beat frequency, the NMBS can be used to measure strain and regional dilation between diastole (full relaxation) and systole (peak contraction). We observe that even in a region with uniform beat frequency, the monolayer of hESC-CMs show a large degree of variability in the direction of motion and magnitude of expansion or contraction (Fig. 4g, h and Supplementary Movie 8). Plotting the dilation for each grid as a function time (Fig. 4i) confirms that all areas are contracting in synchrony. We observed peaks in expansion (Region #8) of 1.04 and contraction (Region #19) of 0.931 (Fig. 4i), while other areas had relatively small changes (Region #21). It appears that the clusters of hESC-CMs that cause these different dilation patterns are larger than the grid size, as the expansion or contraction typically involves multiple adjacent regions.

For further insight, segments connecting the nodes of the NMBS mesh can be used to determine the strain in the X and Y direction at different regions of the culture (Fig. 4j). Additionally, to analyze the pattern of X, Y, shear, and principle strain during the cardiomyocyte contractions, we performed a more complete strain calculation using the X and Y location for each NMBS node over time and their respective pairwise connections within the mesh segments. After accounting for the initial deformation of the NMBS due to its application onto the cells or tissue (Supplementary Fig. 7a, b), we can extract the X, Y, shear, and principle strain components for each segment of the NMBS as it deforms over time due to underlying cardiomyocyte contraction. At peak systole, the principle strain and direction (P_1_, Supplementary Fig. 7c and Supplementary Movie 9), X strain (E_xx_ horizontal component, Supplementary Fig. 7d), Y strain (E_yy_ vertical component, Supplementary Fig. 7e), and the shear strain (E_xy_ component, Supplementary Fig. 7f) reveal the underlying strain components resulting in the NMBS deformation. The X, Y, and shear strain components were also plotted as a function of time for each grid of the NMBS throughout the contraction cycle similar to what was shown for the regional dilation analysis (Supplementary Fig. 7d–f bottom).

Finally, to determine fractional shortening during hESC-CM contraction, we calculated the percent change in area between diastole and systole for a combined region (Region #13, 14, 18, and 19). The mean fractional shortening following three contractile cycles was 3.84% ± 0.06% (Fig. 4k). This measurement is the deformation of the apical surface of the hESC-CMs, as the basal surface is adhered to the coverslip. These data demonstrate the versatility of the NMBS for quantifying heterogeneity in the contractility of hESC-CMs that would not be evident from voltage or calcium imaging alone.

### Mapping 3D strain in developing tissue

**Fig. 5:**
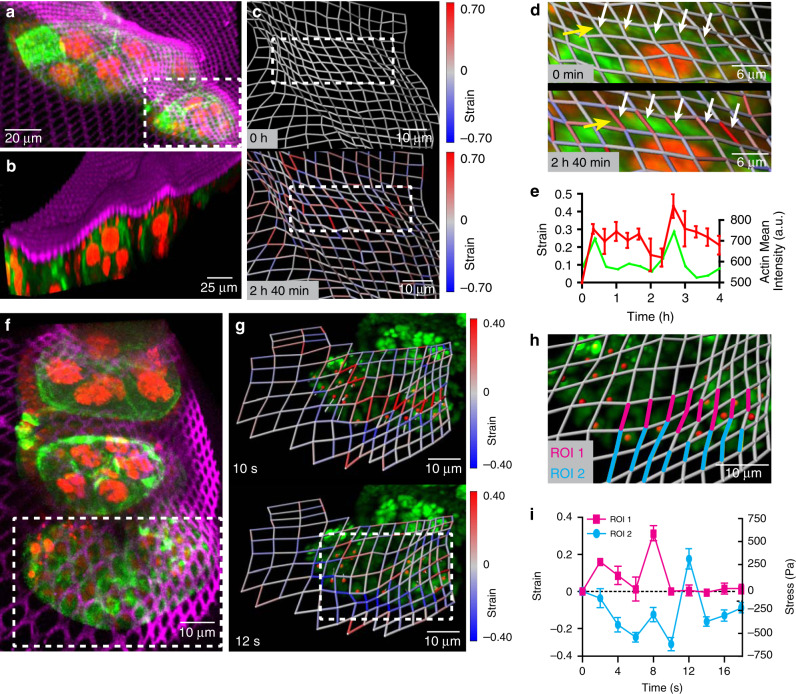
Utilizing the NMBS for 3D strain mapping on *Drosophila* ovariole tissue. **a** Maximum intensity projection of fluorescence image from live 3D confocal imaging of Alexa-647 fibronectin NMBS (Magenta, 2 µm × 2 µm × 2 µm) applied to a developing *Drosophila* ovariole expressing moesin-GFP (Green) and Histone-2B-RFP (Red). **b** 3D render of a diagonal cut plane demonstrating the ability of the NMBS to conform to the 3D surface of the ovariole. **c** Strain analysis and quantification of region in **a** (white box) at two time points show regionally localized tensile (red) and compressive (blue) strain over time. **d** Magnified images (white box in **c**) of high tensile region (white arrows) corresponding to localized moesin-GFP fluorescence (green, yellow arrows). **e** Quantification of the average tensile strain (red, white arrows) within the magnified region shows strain peaks correlate with increased moesin-GFP fluorescence (green, yellow arrows) over a 4-h time course (mean S.D.; *n* = 5 segments over 1 experiment). **f** Maximum intensity projection of fluorescence image from live high-speed spinning-disk confocal imaging of Alexa-647 fibronectin NMBS (Magenta, 2 µm × 2 µm × 2 µm) applied to a contracting *Drosophila* ovariole expressing moesin-GFP (green) and Histone-2B-RFP (red). **g** Time-lapse imaging of region in **f** (white box) at 0.5 Hz of a 20 µm section of a contracting ovariole with the NMBS (magenta) and histone-2B-RFP (green). Regions of tension (*t* = 10 s, max of 1.55) and compression (*t* = 12 s, max of 0.63) are observed during contraction and extension respectively. As the nuclei translate upward during ovariole contraction (*t* = 10 s; red dots, gray commit tails) there is an increase in tension at their leading edge (max tension = 1.44). As the nuclei move down and the ovariole compresses (*t* = 12 s), a band of compression along the bottom edge of the ovariole is observed (max compression = 0.60). **h** Magnified image of strain analysis showing the 3D deformation during ovariole contractions and highlighting regions of compression and tension for quantification. **i** Quantification of the maximum tensile and compressive regions within the strain map, (ROI 1, magenta; ROI 2, cyan) of the contracting ovariole. Stress during contraction was calculated by using values for the elastic modulus of the germarium and stage 1 ovariole of 2 kPa (mean ± S.D.; ROI 1 *n* = 10 segments and ROI 2 *n*  = 14 segments over 1 experiment).

Developing tissues undergoes dynamic changes during morphogenesis as a result of cell-generated mechanical forces. Studying these changes in a direct manner is difficult without interfering with the internal structure and function of the tissue. The NMBS offers a solution to this problem through application of a thin FN mesh onto the surface. To demonstrate this, we used the *Drosophila* ovariole as a developmental model to investigate two aspects of mechanical strain; slow dynamics associated with cytoskeletal rearrangements, and fast oscillations associated with muscular contractions^42^. Ovarioles expressing moesin-GFP to visualize the actin cytoskeleton and histone-2B-RFP to visualize the nucleus were dissected for germarium through stage 6 ovariole chambers^43^, transferred to a glass coverslip, and an NMBS with 2 µm wide lines at 2 µm spacing was applied (Fig. 5a). The NMBS adhered to the 3D surface topology of the ovarioles and made conformal contact, based on cross-sections from confocal images (Fig. 5b and Supplementary Movie 10). A 4-h time-lapse of a stage 4 ovariole (region indicated by dashed white box in Fig. 5a) showed changes in strain magnitude and direction over time (Supplementary Movie 11). At both 20 min and 2 h and 40 min into the imaging series, a large band of tensile strain appeared along the top edge of the stage 4 ovariole (Fig. 5c and Supplementary Movie 11). Closer investigation into this region (dashed white box in Fig. 5c) showed an increase in moesin-GFP intensity associated with the increase in tensile strain (Fig. 5d). The average strain for multiple segments in this region (white arrow heads in Fig. 5d) and moesin-GFP fluorescence intensity plotted as a function of time, revealed a peak in moesin-GFP fluorescence that corresponded to a peak in tensile strain that steadily decreased until the next peak in moesin-GFP fluorescence (Fig. 5e). Thus, we were able to observe correlation between strain dynamics of the NMBS and the assembly of the actin cytoskeleton.

We also used the NMBS to track fast dynamic strains over short time scales with high-speed spinning disk 3D confocal imaging (Fig. 5f). Our goal was to measure the magnitude of tensile and compressive strain during contractions and correlate the peaks in strain with the magnitude and direction of nuclear motion. Analysis of the NMBS region covering the germarium through stage 4 ovariole revealed distinct patterns of tensile (Fig. 5g, top) and compressive (Fig. 5g, bottom) strain (Supplementary Movie 12). These peaks in strain were correlated with the coordinated nuclear movement accompanying the muscle sheath contraction (Supplementary Fig. 8 and Supplementary Movie 12). Quantification of the regions comprising peak tensile (ROI 1) and compressive strain (ROI 2) demonstrated maximum tensile strain of 32.88 ± 8.53% and a maximum compressive strain of 26.73 ± 9.14% (Fig. 5h, i) during a contraction. Additionally, by estimating the elastic modulus of the stage 4 ovariole as 2 kPa^44^, we calculated a peak tensile stress of 657.6 ± 170.6 Pa and peak compressive stress of 534.6 ± 182.8 Pa that the muscle contractions exert on the ovariole. Additional experiments looking at the frequency of ovariole muscular contractions confirmed a contraction frequency of 1 per 12 s (0.083 Hz) and exhibited similar maximum tensile and compressive strain during contractions of 42.95 ± 9.43% and 22.63 ± 8.19%, respectively (Supplementary Fig. 8 and Supplementary Movie 13).

## Discussion

Our results demonstrate that an ultra-thin, fluorescently-labeled FN mesh with tunable dimensions can serve as a NMBS to quantify and map strain from subcellular to multicellular length scales. The NMBS can be directly applied to the surface of a range of biological and non-biological materials acting as a fiducial marker for mapping strain on a surface in 3D space and quantifying the deformation over time. Additionally, by choosing the appropriate fluorophore to conjugate to the NMBS, it can be combined with additional fluorescent biosensors and proteins, allowing for simultaneous monitoring of biological processes and strain measurements.

The NMBS resolution and geometric pattern can be tuned to fit a range of experimental conditions. Our design consisted of a square lattice mesh 1 cm^2^ in overall area, providing adequate coverage for many biological samples. However, this can be scaled up or down to suit a particular need and other patterns (e.g., circular holes, hexagonal arrays, triangular mesh, auxetic lattices) can be used to optimize or enhance fabrication, detection, and analysis in a tissue-specific manner^31^. In this paper, we choose to focus on the square lattice template as a proof-of-principle and will investigate additional NMBS designs in future work.

In this technique there are two aspects that define the strain tracking sensitivity (i) the resolution limits of the NMBS that are dictated by the SIA fabrication capabilities, and (ii) the accuracy of strain tracking that is limited by experimental and optical imaging constraints. In terms of resolution, our results show that a NMBS with mesh dimensions of 2 µm line width and 2 µm spacing enables subcellular measurement of strain (Fig. 3 and Supplementary Fig. 4). At this size, we maintain robust fabrication of the NMBS using SIA while also providing a strain map with single micron scale resolution. However, it is possible to create a NMBS with features below 2 µm using more advanced nanofabrication facilities. For example, SIA has been used to fabricate, FN lines ranging from 100 to 500 nm in width, suggesting that it is technically feasible to pattern an NMBS with features at this scale^30^. In addition to the NMBS fabrication capability and segment resolution, strain tracking sensitivity is determined by the optical imaging platform. In X and Y the resolution limit is ~250 nm (λ/2) and is governed by the microscope objective magnification, numerical aperture, refractive index of the medium, camera pixel size, and the wavelength of light being emitted from a fluorophore^45,46^. As the NMBS is ~4 nm in thickness, the out of plane Z-axis resolution is also determined by the optical configuration, but at best is considered to be more than twice the X–Y resolution and can be estimated to be ~600 nm^45,46^.

In our experiments using the 2 µm by 2 µm NMBS, we performed high-speed, high-resolution spinning disk confocal imaging of contracting *Drosophila* ovarioles at or beyond Nyquist resolution in X, Y, and Z for our system to approach the optical resolution limits of light microscopy (Fig. 5). If additional increases in resolution are needed to resolve smaller changes in NMBS displacement or smaller NMBS mesh dimensions one could measure the point spread function for their imaging platform, oversample in X, Y, and Z and implement conventional deconvolution approaches during post processing and analysis to achieve axial and Z-axis resolutions approaching 50 nm and 200 nm respectively^47,48^. It should also be possible to label the NMBS with a dye such as Alexa Fluor^TM^ 647, which is used in super resolution imaging techniques such as stochastic optical reconstruction microscopy (STORM) to achieve a resolution of ~20 nm^49^. Like all approaches, however, there is a balance between achieving the highest resolution versus the required resolution to answer a particular scientific question. In our studies, we found mesh lattices with 10 µm wide lines and 100 µm spacing to be a good compromise between resolution and coverage area for multicellular tracking of myoblast migration and CM contractility, and 2 µm wide lines and 2 µm spacing to track single cell motility and contracting ovarioles.

Additionally, by creating a strain sensor composed entirely of ECM proteins such as FN, there is no exogenous material restricting cell growth or migration. For cells and tissues that do not express appropriate integrins for FN binding, alternative ECM proteins can be conjugated with a fluorescent dye and used for NMBS fabrication. Aside from the ability to construct the FN NMBS, the SIA process allows for microcontact printing of nanometer thick collagen I, IV, and laminin^31,50,51^. However, FN is an ideal ECM protein for NMBS fabrication because it is hyperelastic and can achieve strains over 700%, making it appropriate for fiducial tracking of large deformations associated with tissue formation^52^. By passively stretching with the underlying cells or tissue, the FN NMBS appears to contribute minimal disruption of developmental processes and native biomechanics. Additionally, as the FN NMBS is thin at ~4 nm (Fig. 1k), it is unlikely to mechanically influence cells adhered to a rigid glass or plastic substrate or 3D multicellular tissues where there is typically a collagen containing ECM matrix that is significantly stiffer and more extensive than the NMBS mesh^53^. Thus, as the cells in 2D and 3D migrate, differentiate, and contract, the FN NMBS is easily deformed to visualize dynamic 3D surface strain.

While we know micropatterned FN can bind to integrins^54^, we do not believe that integrins are required for NMBS adhesion to cells or tissue, and that adhesion can also occur via hydrophobic and electrostatic interactions^55^. Our results show a range of substrates that the FN-NMBS can adhere to such as glass, PDMS, fibrin gels, cell surfaces, and ovariole tissue. While, it is possible that the binding of the NMBS to integrins and subsequent integrin membrane mobility on the cell or tissue surface could restrict or alter fiducial strain tracking, we have not observed this phenomenon. In our experiments, the NMBS segments appear stably associated with the apical surface of cells for up to 24 h (Fig. 3e–k). We did not observe any noticeable degradation of the NMBS by matrix metalloproteinases (MMPs), other proteases, or due to ECM remodeling; however, at longer time points these effects could be more prominent. In cells demonstrating little movement or differentiation, the NMBS remains minimally deformed and bound to its original location on the cell membrane (Fig. 3a–c). In C2C12 cells following induced differentiation, we did not observe binding and unbinding events of the NMBS over the 24-hour time course of imaging. It is, however, still possible that in some circumstances a cell or tissue could become unbound to the NMBS. Additionally, the direction and magnitude of cell migration strongly correlated with the strain observed via NMBS analysis (Supplementary Fig. 5a, b) suggesting that the NMBS is reporting the changes in cell shape and movement within the cell monolayer rather than being transited along the membrane via integrin mobility or displacing due to binding and unbinding events.

Another potential concern with using an ECM-based fiducial marker is the induction of mechanosignaling due to the NMBS application during the experiment. While we cannot rule out potential short-term alterations in mechanosignaling, we did not notice any long-term effects following NMBS application or subsequent imaging (Fig. 3e–k). Cell tracking data (Supplementary Fig. 5a, b) also demonstrated that cells bound to the NMBS were indistinguishable in behavior to the unbound cells. Additionally, it has been shown that cells in tissue culture can secrete and assemble fibronectin-rich ECM that is typically far more dense than the fibronectin present in the NMBS^56^. While we did not stain for total fibronectin in our experiments, the presence of cell secreted fibronectin is well established in the literature^56–58^. Thus, the NMBS represents a small fraction of the total fibronectin in the cell culture. This is also true for applications to ovariole tissue where the FN-NMBS would represent a minor component of the overall tissue ECM (Fig. 5a–e). Future work will be needed to comprehensively determine any role the NMBS plays in mechanotransduction during tissue development.

In developmental biology, measurement and identification of mechanical forces required for tissue formation are often limited to 2D indirect approaches and invasive or destructive techniques^23,24^. Recent advances in high speed confocal fluorescence imaging techniques have facilitated the development of 3D biomechanical force sensors such as 3D traction force microscopy and functionalized oil microdroplets^21,27^. 3D traction force microscopy is an elegant solution for directly measuring subcellular strain within a substrate of known material properties, but analysis is computationally demanding and is difficult to extend beyond the cellular length scale. Additionally, traction force microscopy would be difficult to use on the free apical surface of an unembedded cell. Uniformly patterning fluorescent microbeads along the apical cell surface would be challenging and would likely require functionalization to facilitate bead surface adhesion. Furthermore, without a matrix to embed the beads in, measurements would be limited to strain, and the functionalized beads could be taken up by the cell, depending on their size. For functionalized oil microdroplets the sensor resolution depends on the size and deformability of the droplet to provide direct quantification of the intercellular mechanical force within a tissue, but sparse labeling is needed to avoid developmental tissue defects. The ability for the NMBS to directly measure surface strain over a spatial range from approximately 1 μm to 1 mm in biologically relevant hydrogels and tissue samples serves to bridge this gap and complement these modalities. Application onto isolated *Drosophila* ovarioles highlights the ability of the NMBS to track 3D surface tissue strain over time with cellular resolution during development, as well as throughout fast oscillating muscle sheath contractions. One can imagine that by combining fluorescence imaging-based approaches such as 2D traction force microscopy (cell-substrate interface), functionalized oil microdroplets (intercellular), and the NMBS (free apical surface), we could simultaneously map true 3D biomechanical strain to better understand how force is transmitted within cells and tissue. In future work, we plan to implement these combined approaches to study compaction forces during engineered cardiac tissue maturation and evaluate the changes in surface strain accompanying tissue expansion and contraction for many organ systems (i.e., bladder, heart, kidney, and blood vessels).

Currently, the NMBS platform maps strain by determining a change in length or area between geometric regions defined by NMBS mesh nodes and can approximate stress from strain if the elastic modulus of the cells or tissue is known. This analysis is highly informative, but there are more advanced strain analysis and deformation tracking techniques such as digital image correlation and direct deformation estimation^59–61^. Fortunately, the information required to calculate a tensorial strain state based on the strains of the edges of the NMBS mesh shape is inherently captured in our data and was demonstrated in two dimensions as X, Y, shear, and principle strain for each NMBS region for the contractile cardiomyocytes (Supplementary Fig. 7). Additionally, if a dense (2 µm or smaller) NMBS mesh pattern was chosen, the complete continuum of the surface of the cell or tissue could be estimated using interpolation between adjacent nodes in the NMBS^62^. This would allow for not only a complete strain analysis of the surface in 3D but also a measurement of the energy in the surface^63^. Combined with a constitutive law for the NMBS, changes in strain energy could be derived from this continuum approximation^64^, which would allow us to calculate stresses at the surface of the NMBS in contact with the cells or tissue. In future work, we plan to move beyond node tracking and improve the NMBS tracking with more sophisticated point-cloud analysis that incorporates information about the shape of each segment in the NMBS. This will allow for a more detailed analysis of the strains and a better understanding of the stresses driving processes such as developmental morphogenesis and disease progression.

## Methods

### Fabrication of the nanomechanical biosensors

A step-by-step protocol describing the fabrication process can be found at Protocol Exchange^65^. The NMBS were fabricated using an adaptation the surface-initiated assembly technique (SIA)^31,66^. Briefly, square-lattice meshes (e.g., 100 μm length × 100 μm width × 10 μm line thickness per grid segment) were first designed using AutoCAD software. The CAD file was then transferred to a transparency photomask (CAD/Art Services, Inc., Bandon, OR, USA), where the spaces and the segments of the square-lattice meshes were dark and transparent, respectively (Fig. 1a). Square glass wafers (Fisher No. 12-543-F 45 × 50 × 2 mm) were spin-coated with Photoresist SPR-220.3 at 5000 rpm for 20 s, baked on a 115 °C hot plate for 90 s, exposed to ultraviolet (UV) light through the transparency photomask, baked on a 115 °C hot plate for 90 s, and finally developed for 1 min using MF-26A developer. Sylgard 184 (Dow Corning) polydimethylsiloxane (PDMS) elastomer was prepared per manufacturer’s directions by mixing 10 parts base to 1 part curing agent (by weight) using a Thinky-Conditioning mixer (Phoenix Equipment Inc., Rochester, NY, USA) for 2 min at 2000 rpm followed by 2 min of defoaming at 2000 rpm. The PDMS was then cast over the topographically-patterned photoresist-coated glass wafer inside a petri dish and placed in a 65 °C oven to cure the PDMS (Fig. 1b). Square PDMS stamps ~1 cm^2^ were cut out of the ~5 mm thick PDMS layer.

For the SIA process, the PDMS stamps were sonicated in 50% ethanol for 30 min, dried with nitrogen gas, and coated with a 50 μg/mL human FN solution (Corning). The FN solution contained a 40% Alexa Fluor® 555, or 633 (ThermoFisher) C_5_ fluorescently-labeled FN. After 1 h of incubation at room temperature (Fig. 1c), the FN-coated PDMS stamps were rinsed in sterile ddH_2_O, dried with nitrogen, and stamped onto 25 mm circular glass coverslips (Fisher No. 12-545-86 25CIR-1D). Prior to stamping, the coverslips were cleaned and spin-coated at 6000 rpm for 1 min and 37 sec with 2% g/mL poly(N-isopropylacrylamide) (PIPAAm) (Scientific Polymer, mw 300,000) diluted in 1-butanol (Fig. 1d). After 1 h the PDMS stamps were carefully removed from the glass coverslips, leaving behind a microcontact-printed fluorescently-labeled FN square-lattice mesh on the sacrificial PIPAAm surface prior to release and subsequent transfer to different materials (Fig. 1e). Design and fabrication of the 20 μm length × 20 μm width × 10 μm line thickness NMBS and 2 μm length × 2 μm width × 2 μm line thickness NMBS was identical to the above-mentioned procedure.

### Atomic force microscopy

Atomic force microscopy (AFM MFP3D–Bio, Asylum Research) was used to determine the thickness of the fibronectin lattice structure that comprises the NMBS. Alexa-555-FN NMBS (2 µm by 2 µm square lattice with 2 µm thick FN filaments) were patterned onto PIPAAm-coated glass coverslips. The FN-NMBS was scanned using AC mode with AC160TS cantilevers (Olympus Corporation) and the Asylum Research AFM Software version 14.23.153. A scan area of 20 µm by 20 µm was chosen to get a sufficient representation of the NMBS thickness. Line profiles of the height trace were obtained using the IGOR Pro 6.1 software (WaveMetrics) from the AFM height channel. Thickness was determined by calculating the difference between the minimum and maximum height from the line profiles. Peaks within the height trace denote the NMBS FN and the valleys represent the void space within the NMBS lattice. The spacing between the NMBS features also confirms the fabrication dimensions and accuracy.

### Creation of test strips for uniaxial testing

For uniaxial testing, NMBS were integrated onto dog bone shaped tensile testing strips made of fibrin and PDMS elastomer and subjected to uniaxial mechanical strain. See Supplementary Fig. 1 for dimensions of dog bone test strips which have been slightly adjusted from ASTM E8 to fit our experimental setup.

### Fibrin test strips

For fibrin test strip fabrication, 40 mg/mL bovine fibrinogen (MP Biomedicals) was combined with 10 U/mL bovine thrombin (MP Biomedicals) at a final concentration of 36 mg/mL and 1 U/mL, respectively, to catalyze the conversion of fibrinogen to fibrin^67^. The fibrin mixture was casted into 3D printed plastic dog bone molds containing small Velcro pieces (VELCRO® Super-Grip Double-Head Hook) inserted at both ends to grip the gel during tensile testing. Molds were sprayed with Teflon (WD-40® Specialist_TM_ Dirt & Dust Resistant Dry Lube Spray) to facilitate mold release following casting. After 30 min at room temperature, the fibrin hydrogel solidified.

### PDMS test strips

For PDMS test strip fabrication, Sylgard 184 and Sylgard 527 (Dow Corning) were blended at a mass ratio of 5:1^50^. Briefly, Sylgard 184 and 527 were prepared per manufacturer’s directions and mixed in a Thinky-Conditioning mixer for 2 min at 2000 rpm followed by 2 min of defoaming at 2000 rpm. Once mixed, the 5:1 PDMS 184:527 was casted into 3D printed dog bone molds. The molds were placed in a 65 °C oven overnight to ensure proper PDMS curing. Prior to NMBS application, the PDMS test strips were treated with 15 min of UV Ozone to create a hydrophilic surface to facilitate NMBS attachment.

### Transferring NMBS to test strips

To transfer the NMBS to the hydrogel test strips, the hydrogel test strip was pressed onto the PIPAAm-coated glass coverslip stamped with the fluorescent NMBS. Warm (37 °C) 1× PBS was added to the glass coverslip and test strip. As the temperature dropped below the lower critical solution temperature of PIPAAm (~32 °C), the gradual dissolution of PIPAAm resulted in release of the coverslip and integration of the NMBS onto the bottom surface of the hydrogel test strip (Fig. 1f, g).

### Uniaxial tensile testing

Fibrin and PDMS test strips containing an applied Alexa-555-FN-NMBS were mounted to micromanipulators (Eppendorf TransferMan NK) at both ends using fast hardening two-part epoxy (Devcon) through the embedded Velcro attachment points (Fig. 2a). Uniaxial testing was performed on top of a Nikon Eclipse TI wide-field fluorescence microscope. Fluorescence imaging was performed with a CoolSnapES (Photometrics) camera, X-Cite 120PC light source (Excelitas Inc.), and appropriate excitation and emission filters for Tx-Red fluorescence. Fiducial marks spaced 8 mm apart were introduced in the gauge region of the test strip on the top surface to provide tracking of macroscopic deformation (Fig. 2b). Uniaxial tensile testing was performed in 100 μm increments. Fluorescence images of the NMBS (10× air objective) and macroscopic images (GoPro HERO3 camera) of fiducial marks were acquired at each step. Macroimaging of the test strip side edges provided tracking of compressive strain ε_2_ at the macroscopic scale. Differential interference contrast (DIC) imaging of the test strip top and bottom surface provided thickness measurements during mechanical testing for calculation of macroscopic compressive strain ε_3_ along the z-axis. For uniaxial testing involving the circular defect, a circular defect of diameter 0.4 mm was introduced in the center of a 555-FN-NMBS PDMS test strip gauge region using a hole punch tool (Fig. 2k). Test strips with circular defects were mounted onto micromanipulators for uniaxial testing.

### 2D Image analysis for quantification of NMBS strains

MATLAB-based analysis code was written to measure the changes in NMBS segment length from the imaging data acquired during uniaxial tensile tests to calculate the NMBS microscopic 2D strains. Figure 2d shows a representative microscopy image of NMBS at macroscopic tensile strain of PDMS equal to 0.45. First, the image series was imported into MATLAB, background subtracted, and thresholded to convert the NMBS fluorescence into a binary image. The binary image was skeletonized to identify the intersection nodes. Following node-to-node pair assignment lengths between each node pair was calculated and overlaid onto the original NMBS image (Fig. 2e). To quantify strain during uniaxial testing, the segment lengths between node pairs were calculated for each testing interval and converted into engineering strains ($\varepsilon = \frac{l}{{l_0}} - 1$) with respect to their undeformed reference length. Positive values of strain corresponded to tensile components of strain, while negative values of strain corresponded to compressive components of strain (Fig. 2f). To visualize the strain fields, we converted the strain values for each segment into a color-coded map for both tensile and compressive mechanical strains of NMBS (Fig. 2f).

### ANSYS mechanical simulations

To validate the strain results obtained by the NMBS during uniaxial tensile testing we performed FEA to simulate the tensile and compressive strains for a PDMS strip with a 0.4 mm circular defect (Fig. 2n). Fusion 360 (Autodesk) modeling software was employed to create an in-silico representation of the tested PDMS strip with the circular defect. Of note, the model was made longer than the sample tested on the stretcher. This was done to negate the edge effects of having a fixed boundary condition. Once modeled, the object was exported as an.STL and imported into the 2019 ANSYS Mechanical static structural simulation.

A Mooney-Rivlin 2 parameter model was assigned to the object. The general form of this model is $W = C_1\left( {I_1 - 3} \right) + C_2\left( {I_2 - 3} \right)$ where *W* is the energy strain function, *I*_1_ and *I*_2_ are the first and second invariants of the Lagrange-Green deformation tensor. Furthermore, the material was assumed to be isotropic. *C*_1_ and *C*_2_ were assigned values of 0.0213 MPa and 0.0021 MPa, respectively for PDMS with a 5:1 mass ratio of Sylgard 184:527^50^. These parameters were determined by fitting experimental uniaxial stress-strain curves from a solid strip of our PDMS. An incompressibility parameter of 0.0001 MPa^−1^ was assigned to enforce isochoric behavior of the model. A fixed boundary condition was applied to one face of the object. On the opposite face, a displacement boundary condition was set to produce a 60% strain of the entire sample, recapitulating the maximum strain applied during experimental uniaxial testing. After the simulation converged, global short axis (X) and long axis (Y) strains were calculated and rendered (Fig. 2n).

### Preparation of sterile gelatin carriers

Gelatin type A carriers (20% w/v) were prepared by casting into a circular silicon mold. The silicon molds were removed, and the gelatin gels were UV treated for 10 min to sterilize. NMBS-patterned PIPAAm-coated glass coverslips were placed with NMBS facing down onto the gelatin carriers inside the cell-culture hood for 1 min. Sterile room temperature ddH_2_O was added between the glass coverslips and the gelatin carrier to cause dissolution of the PIPAAm and subsequent release and transfer of the NMBS to the top surface of the gelatin (Fig. 1f). Following transfer, all remaining water surrounding the NMBS-gelatin carrier was aspirated. The NMBS-gelatin carrier was stored in a sealed container at 4 °C until ready for tissue application.

### Application of the NMBS onto cells

Cells were grown on a glass coverslip and transferred to a metal imaging chamber (Attofluor Cell Chamber, ThermoFisher #A7816). Before the addition of media, the NMBS-gelatin carrier was lifted and applied to the cells with the NMBS side down. Light pressure was applied to the gelatin carrier to fully adhere the NMBS to the cells. Subsequent incubation at 37 °C for 5 min melted the gelatin and integrated the NMBS onto the cells (Fig. 1h). Without aspirating the melted gelatin, fresh cell culture media was added and incubated at 37 °C for 5 min. Prior to imaging, the media was changed to remove the melted gelatin and label cells with CellTracker™ Green CMFDA Dye (Invitrogen) and 2 μg/mL Hoechst 33342 dye (ThermoFisher Scientific) to stain for the cell nuclei (Fig. 3b). A final media change was performed to phenol red free CO_2_ independent Lebovitz L-15 (Invitrogen) media containing appropriate serum and nutrients for each cell line.

### Application of the NMBS onto living tissue

For application to *Drosophila* ovariole tissue, the ovarioles were fanned out onto a glass coverslip and allowed to partially dry. The coverslips were then transferred to a metal imaging chamber (Attofluor Cell Chamber, ThermoFisher #A7816) and the NMBS-gelatin carrier was lifted and applied to the top side of the ovarioles with the NMBS side down. Light pressure was applied to the gelatin carrier to fully adhere the NMBS to the ovariole surface. Initial incubation at 37 °C for 3 min was followed by incubation at 28 °C for 10 min in Schneider’s media containing Hoechst 33342 to melt the gelatin and integrate the NMBS onto the top surface of the ovarioles (Fig. 5). A final media change was performed with fresh phenol-red free Schneider’s media before imaging at 25 °C.

### Cell lines and cell culture

For our investigations of cellular strain using the NMBS we used human skeletal muscle cells (HSMC, Cook MyoSite), C2C12 mouse myoblast cells (CRL-1772, ATCC), and HES3 human embryonic stem cell (hESC)-derived cardiomyocytes. The HSMCs were grown in MyoTonic Growth Medium Kit (MK-4444, Cook MyoSite) with 1% (v/v) penicillin–streptomycin (15140-122, Life Technologies) at 37 °C with 5% CO_2_ and maintained at ≤50% confluency following the manufacturers recommended protocol^68^. Prior to NMBS application and live fluorescence imaging, HSMCs were plated onto 25 mm glass coverslips and allowed to grow for 24 h.

Culture and passage of C2C12 cells was performed in DMEM-high glucose (15-013-CM, Corning CellGro) supplemented with 10% (v/v) fetal bovine serum (FBS) (89510-186, VWR), 1% (v/v) L-glutamine (25030-081, Life Technologies), and 1% (v/v) penicillin–streptomycin (15140-122, Life Technologies) at 37 °C with 10% CO_2_ and maintained at ≤80% confluency. Prior to NMBS application and live fluorescence imaging, C2C12 cells were plated onto 25 mm glass coverslips, and allowed to grow for 24 h. To begin myoblast differentiation and myotube formation, cells were transitioned to medium consisting of DMEM-high glucose supplemented with 2% (v/v) heat-inactivated horse serum (H1138 Sigma-Aldrich), 1% (v/v) L-glutamine (25030-081, Life Technologies), and 1% (v/v) penicillin–streptomycin (15140-122, Life Technologies).

Human cardiomyocytes derived from HES3 human embryonic stem cells (obtained from ESI and WiCell Cat. No. ES03) were cultured and differentiated according to established and published protocols^69,70^. Briefly, hESCs were grown in Essential 8 (E8) medium (A1517001, Life Technologies) supplemented (S1459, Selleck Chemicals) in 6-well plates coated with Geltrex (12 µg/mL, A1413301, Life Technologies). Once 50% confluency was achieved, cardiomyocyte differentiation was initiated^69,70^. On day 0, RPMI/B27 with 6 µM CHIR99021 (C-6556, LC laboratories) media was added. On day 2, the media was replaced with RPMI/B27 with 2 µM Wnt-C59 (S7037, Selleck Chemicals). On day 4 and 6, the media was replaced with fresh RPMI/B27. On day 8 and 10, the media was replaced with CDM3. On day 12, lactate-supplemented media (CDM3L) was used for metabolic purification of spontaneously beating cardiomyocytes. Beating cardiomyocytes were washed with 1X PBS and detached from the surface with TrypLE express for 15 min at 37 °C. Detached cells were pipetted into DMEM/F12 media and centrifuged at 200 × *g* for 7 min. Purified cardiomyocytes were seeded on 12 µg/cm^2^ Matrigel (356231, Corning)-coated coverslips with CDM3L and purified for 5 days. Following differentiation and lactate-based metabolic purification^71^, spontaneously beating cardiomyocytes were maintained in CDM3 medium for up to 28 days prior to experimentation^72^. Prior to NMBS application and live fluorescence imaging, purified cardiomyocytes were plated onto 25 mm glass coverslips coated with Matrigel (12 µg/cm^2^, 356231, Corning) and maintained in CDM3 medium.

### Live cell fluorescence microscopy

Various confocal fluorescence microscopes were utilized to acquire 3D time-lapse images of the NMBS applied to cells and tissue. For imaging of single cells (HSMCs, Fig. 3a) and *Drosophila* ovariole growth (Fig. 5a) we performed imaging on an upright Nikon FN1 resonant scanning confocal microscope with a 40X CFI Apo NIR W objective, 4 laser lines (405, 488, 555, 633), 4 internal detectors (2 PMTs, 2 GaAsP), and a heated stage platform to maintain the temperature at 37 °C. For live imaging of *Drosophila* ovarioles the temperature was maintained at 28 °C. 3D image stacks were acquired for each time point in NIS Elements using a 1 μm z-step interval. For the HSMCs and *Drosophila* ovarioles, 3D image stacks were acquired every 10 and 20 min respectively for the duration of the experiment.

To evaluate NMBS strain during the initiation of C2C12 cell differentiation (Fig. 3e) we performed live confocal fluorescence imaging on an inverted Zeiss 700 LSM microscope equipped with a 20× Plan Apo objective, 4 laser lines (405, 488, 555, 633), 2 internal spectral PMT detectors, and a live cell incubation system to maintain the temperature at 37 °C and CO_2_ at 5%. 3D image stacks were acquired for each time point in Zeiss Zen software using a 1 μm z-step interval. For the C2C12 cells, 3D image stacks were acquired every 30 min to limit the light exposure for the duration of the 24-h experiment.

In order to visualize and track the 3D strain during *Drosophila* ovariole muscle sheath contractions we utilized spinning disk confocal fluorescence microscopy (Fig. 5f). Specifically, imaging was performed using a XDi spinning disk confocal system (Andor) on a Nikon TiE-2000 inverted microscope with a 40X Apo LWD WI objective. The imaging system also consisted of fully temperature and humidity control to maintain 25 °C, a mechanical Piezo XYZ-stage (Nikon), iXon 897 Ultra EMCCD camera (Andor), a 5 line laser combiner (405, 488, 515, 555, 647; Andor), a Lambda 10-3 filter wheel (Sutter), IQ2 imaging software (Andor), and an active air isolation table (TMC). High speed 3D image stacks were acquired for each time point in IQ2 software using a 0.2 μm z-step interval (Fig. 5g). For the fast contractions, 2 and 3 fluorescence channel 3D image stacks were acquired every 1 s for the duration of the experiment.

### 3D strain quantification and imaris strain mapping

For the image segmentation and strain quantification of the NMBS in 3D we developed an Imaris (Bitplane) and MATLAB (Mathworks) image analysis pipeline^73^. 3D image stacks obtained from confocal fluorescence microscopy were imported into Imaris for rendering and visualization. To identify intersection nodes of the NMBS, we first created a surface object of the NMBS based upon fluorescence intensity and performed a distance transformation. Nodes of the NMBS tend to have a high distance value when looking at the distance transformation within a surface. The Distance transformation was then used as the target image for spot detection in Imaris. Spots were placed at each NMBS intersection first through automated detection and then subsequent manual editing. Spot tracking through time ensured that each node was properly identified and tracked throughout the entire time series. Once the spots were identified at each NMBS intersection node, a MATLAB script was run to initiate node to node pair identification and strain calculation.

We developed a MATLAB-based Imaris Xtension Biomechanical Analysis package to calculate 3D strain from the fluorescence NMBS images. To calculate strain from the change in length between paired NMBS nodes, coordinates for each spot in X, Y, Z were exported and read into MATLAB (XTSpotsToFilamentsWithStrain.m)^73^. An image of the NMBS in its reference state was imported and maximally intensity projected into a 2D reference image for spot overlay. A skeletonized 2D version of the NMBS at time 0 was used as a road map for node pair assignment. We implemented an algorithm to recursively traverse the skeletonized image and connect paired nodes. In the event of improper pair assignment, manual correction is made to either add or remove node pairs. Once all node pairs have been correctly assigned, the 3D distance between each pair is calculated and used for subsequent engineering strain determination.

We next created an import Xtension in Imaris (XTFilamentLengthStrain.m)^73^ to import each segment strain value as a filament object in Imaris that can be color coded with a strain statistic. Filaments were created for all node pairs and tracked for each time point. The change in segment length of the NMBS are normalized around 0. Positive strain values corresponded to tensile strain and negative strain corresponded to compressive strain. Individual or groups of segments can be selected for additional data analysis and quantification as an exported Microsoft Excel data sheet. To determine regional dilation and contraction within the NMBS over time, a binary mask was created from the imported Imaris filaments object for each time point and used to generate a Cell object within the Imaris area. A custom Imaris Xtension was then created to calculate regional dilation and contraction from the change area defined by a Cell within the NMBS grid region (XTCellAreaDilation.m)^73^. Changes in dilation were visualized as a color-coded dilation statistic normalized around 0, with positive dilation values corresponding to expansion and negative dilation corresponding to contraction. All MATLAB scripts and functions as well as Imaris Xtensions have been made freely available and open source through at Zenodo.org (10.5281/zenodo.4065743)^73^. Images of the NMBS strain at selected time points and videos showing 3D changes in strain and dilation throughout time were exported from Imaris using the built-in animation capabilities. For the 2D dilation analysis based on the deformation of the NMBS, a maximum intensity Z-projection of the time-series image stack was performed using ImageJ to create a 2D representation of the NMBS. The NMBS channel was extracted, thresholded, and converted to a binary mask that defined the NMBS regions for all time points. This image time series was imported into Imaris and analyzed with our XTCellAreaDilation.m Xtension^73^. The composite deformation image of the NMBS over time was constructed by merging the time-series images and applying a custom look up table corresponding to each time point.

### Identification of subcellular strain regions and ROIs

Imaris was used to define and quantify subcellular regions of the NMBS during HSMC migration. After creation of the NMBS filament objects within Imaris using the XTFilamentLengthStrain.m Xtension^73^ the user can highlight and select any group or individual filaments for further analysis. We choose to select groups of filaments corresponding to the horizontal (X-axis) and vertical (Y-axis) within regions approximated to be the nucleus, cytoplasm and peripheral membrane regions of the apical surface. Each group was color coded using the label function within Imaris. Using the statistics tab, the individual strain values for each group over all time points can be exported for additional analysis and graphing. This same approach was used for ROI identification and analysis throughout the paper.

### Cell motility tracking

C2C12 cell motility was tracked using the Imaris spots function. Briefly, the CellTracker fluorescence channel was used to define the cell regions over time. Using the spots creation wizard in Imaris, the centroids of the cells were identified and tracked over time using a autoregressive motion tracking algorithm. Manual editing of the spot placement and track identity was performed to ensure that all cells were tracked during the entire time course without gaps. Trajectories were plotted, color coded and visualized using built in functionality.

### Cardiomyocyte beat frequency mapping

To begin, fluorescence images of spontaneously beating and electrically stimulated cardiomyocytes with 555-FN-NMBS applied were acquired via Micro-Manager software on an inverted Nikon Eclipse TI wide-field fluorescence microscope with a Nikon 20× Plan Apo Lambda objective, a Prime 95B Scientific CMOS camera (Photometrics), X-Cite 120PC light source (Excelitas Inc.), and appropriate excitation and emission filters for GFP and Tx-Red fluorescence.

Cardiomyocytes were imaged in Tyrode’s medium containing 5 µM calcium indicator dye Cal 520 AM (21130, AAT Bioquest), and paced at 1 and 2 Hz using two parallel platinum electrodes with an 80 V, 20 ms, square wave pulse applied with a Grass Stimulator. A custom MATLAB code (BeatFrequencyFFTMapping.m)^73^ was created to determine and evaluate the heterogeneity in the beat frequency across a field of cardiomyocytes from the NMBS motion and the fluctuations in calcium indicator dye fluorescence intensity. Time series images were downsampled and gaussian blurred to decrease the effects of noise. To extract the beat frequency information from the fluorescence images, fluorescence intensity over time was measured for each pixel within the image series and smoothed using a Savitsky-Golay filter. A Fast Fourier Transform (FFT) of the smoothed fluorescence intensity over time was performed to extract the dominant frequency for each pixel location from the peak of the power spectral density plot^74^. A pseudo image was then created consisting of the dominant frequency at each pixel location within the image and a custom look up table was utilized to visualize beat frequency heterogeneity. To regain the original image resolution the pseudo image was upsampled resulting in a final frequency map (Fig. 4d). For the NMBS FFT analysis, the final frequency map was pass through filtered with a binary mask of the original NMBS fluorescence intensity to restrict the analysis to the boundaries of the NMBS location at time = 0.

### 2D strain calculations of cardiomyocytes

The images acquired for cardiomyocyte beat frequency analysis were reanalyzed and used to extract the 2D strain components. The tracking and location information (X, Y) for the NMBS intersection nodes were exported from Imaris (Bitplane) as an Excel file. This data was formatted and imported into a custom MATLAB script (totalStrainCode.m)^73^ that calculated the 2D strain tensor relative to the as-applied state as well as the principal strain and direction for each of the square elements of the NMBS at each time point where an image was recorded^75^. This data was output into folders as images of the deformed NMBS with color coded infill corresponding to E_xx_, E_yy_, E_xy_, and P_1_ with principal vectors. Additionally, the data for each square element of the NMBS was plotted through time for visualization of temporal phenomena (Supplementary Fig. 7c–f). Additional methods and instructions for extracting the 2D stain state using this approach can be found in the supplementary software guide.

### *Drosophila* ovariole dissection and isolation

Fly stocks used in this study were *Moesin-GFP/TM6,Tb*^76,77^ and *P{His2Av-mRFP1}III.1* (Blooming *Drosophila* Stock Center #23650). A fly stock containing both Moesin-GFP and H2Av-RFP was generated by crossing the aforementioned parental fly lines (*P{His2Av-mRFP1}III.1, Moesin-GFP/TM6,Tb)*. All fly cultures were reared, and adult progeny maintained at 22–23 °C, 70% relative humidity, and 12-12 hr light-dark cycle. One- to two-week-old adult female flies expressing either Moesin-GFP or a combination of Moesin-GFP and Histone 2A-RFP were transferred to vials containing cornmeal fly food and yeast paste for five days. On the fifth day, ovaries were extracted from the female flies in 22 °C unsupplemented Grace’s media (Thermo, Cat#: 11595030) using standard Dumont forceps. For each set of ovaries, the outer fibrous sheath was removed and individual ovarioles were teased apart. Teased ovaries were kept in 22 °C unsupplemented Grace’s media until ready for mesh application on the same day.

### Statistics and data analysis

Statistical and graphical analyses were performed using Prism 7 (GraphPad) software and Excel (Microsoft v16). Statistical tests were chosen based on the experimental sample size, distribution, and data requirements. In all cases, the relevant reproducibility for evaluating the NMBS as a strain sensor was our ability to apply, detect, and analyze individual segments within the NMBS lattice. Therefore, our use of statistical analysis in this paper was not to determine a statistical difference between two experimental groups, but rather to calculate the reproducibility of measurement for the NMBS within a given experiment. For this reason, all error bars are shown as mean ± standard deviation within a given experiment over time and the *n* values are described as the number of individual lattice segments used for quantification. Statistical analyses of the uniaxial tensile testing results data were plotted as mean ± standard deviation (S.D.) and evaluated for correlation using a Pearson correlation coefficient. For quantification and evaluation of NMBS segmental strain, individual strain segments of interest were selected in Imaris and exported for analysis in Excel (Microsoft). Identified regions showing similar tensile or compressive strain were averaged and plotted in Prism 7 (GraphPad) as mean ± standard deviation. Preparation of figures and visuals were constructed in Adobe Photoshop and Illustrator CS6. Fluorescence images were edited in Fiji (ImageJ NIH) and Imaris 9.5.1 (Bitplane). Advanced image analysis and quantification were performed in MATLAB (Mathworks) using custom supplementary software.

### Reporting summary

Further information on research design is available in the Nature Research Reporting Summary linked to this article.

## Supplementary information

Supplementary Information

Supplemental Movie 1

Supplemental Movie 2

Supplemental Movie 3

Supplemental Movie 4

Supplemental Movie 5

Supplemental Movie 6

Supplemental Movie 7

Supplemental Movie 8

Supplemental Movie 9

Supplemental Movie 10

Supplemental Movie 11

Supplemental Movie 12

Supplemental Movie 13

Description of Additional Supplementary Files

Reporting Summary

## Data Availability

The raw image data used for strain mapping of PDMS, HSMCs, C2C12 cells, and *Drosophila* ovarioles in this paper are available at Zenodo.org (10.5281/zenodo.4064033)^78^. Figures with raw data provided are: Figs. 2–5, along with Supplementary Figs. 2, 6, and 8.

## Code availability

All generated software and analysis packages are open source released under the CC-BY-SA 4.0 license and can be downloaded from Zenodo.org (10.5281/zenodo.4065743)^73^. All versions for custom software are version 1.0. For assistance or questions please refer to the included software guides or contact the corresponding author.
