# Supplementary material for: Fibronectin-based nanomechanical biosensors to map 3D surface strains in live cells and tissue
Source: Nat Commun. 2020 Nov 18;11:5883. doi: 10.1038/s41467-020-19659-z (PMC7675982; doi:10.1038/s41467-020-19659-z)
Supplement: Supplementary file 1 — Supplementary Information [file 41467_2020_19659_MOESM1_ESM.pdf]

Fibronectin-Based Nanomechanical Biosensors to Map 3D Surface Strains in Live Cells and Tissue

Supplementary Information:

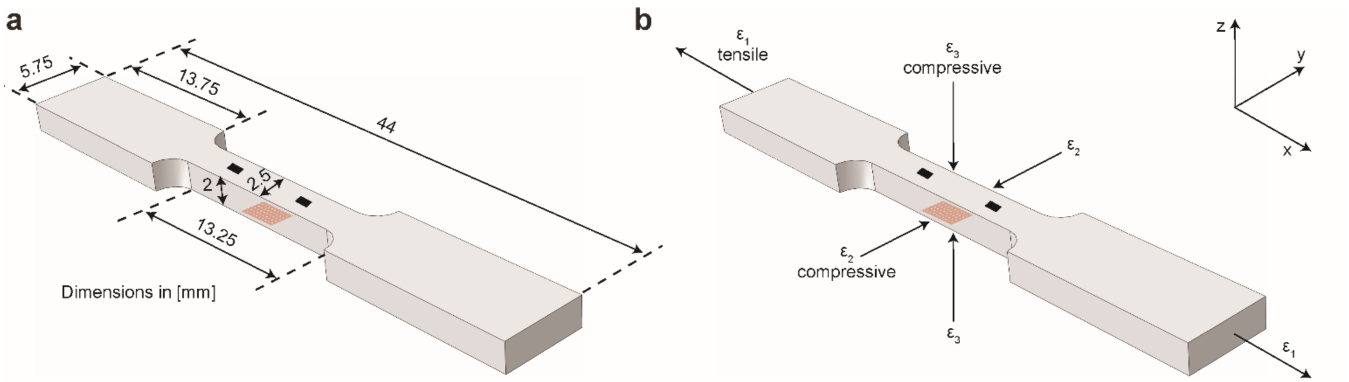

Supplementary Fig. 1 | Design and dimensions of tensile testing strip. a. The overall design of the tensile testing strip was chosen to mimic a classic "dog bone" structure commonly used for uniaxial mechanical testing. The NMBS was applied to the underneath side of the hydrogel test strip and macro fiduciary marks were applied to the top surface for tracking during tensile testing. **b.** Mechanical strains were defined as ε_1 (tensile, X, in the direction of elongation), ε_2 (compressive, Y, perpendicular to the direction of elongation), and ε_3 (compressive, Z, orthogonal to the XY axis).

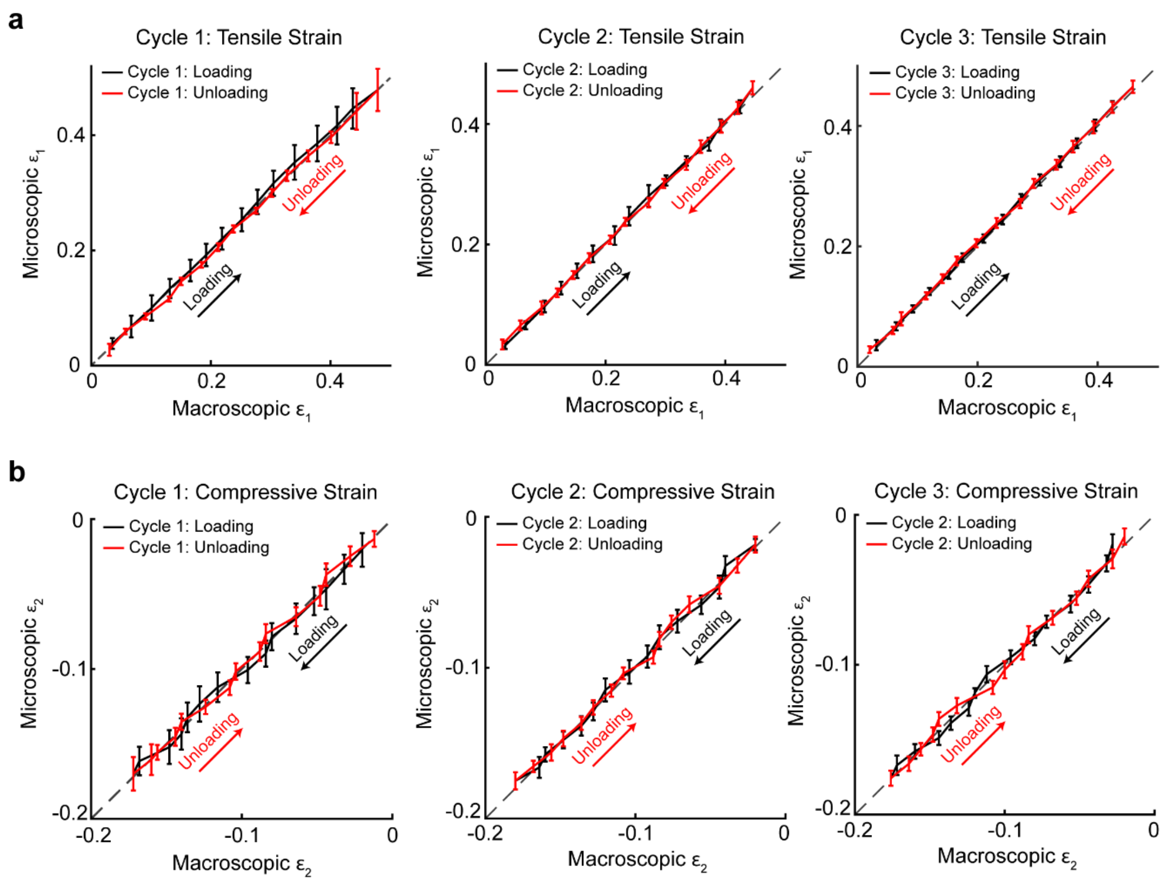

Supplementary Fig. 2 | Measuring microscopic PDMS strain with the NMBS during uniaxial cyclic loading. a. Results from three cycles of cyclic loading (black = loading; red = unloading) showing accurate NMBS tensile strain ε_1 tracking during loading and unloading (mean ± S.D.; n = 12 segments over 1 cycle for each graph). **b.** Results from three cycles of cyclic loading (black = loading; red = unloading) showing accurate NMBS compressive strain ε_2 tracking during loading and unloading (mean ± S.D; n = 12 segments over 1 cycle for each graph).

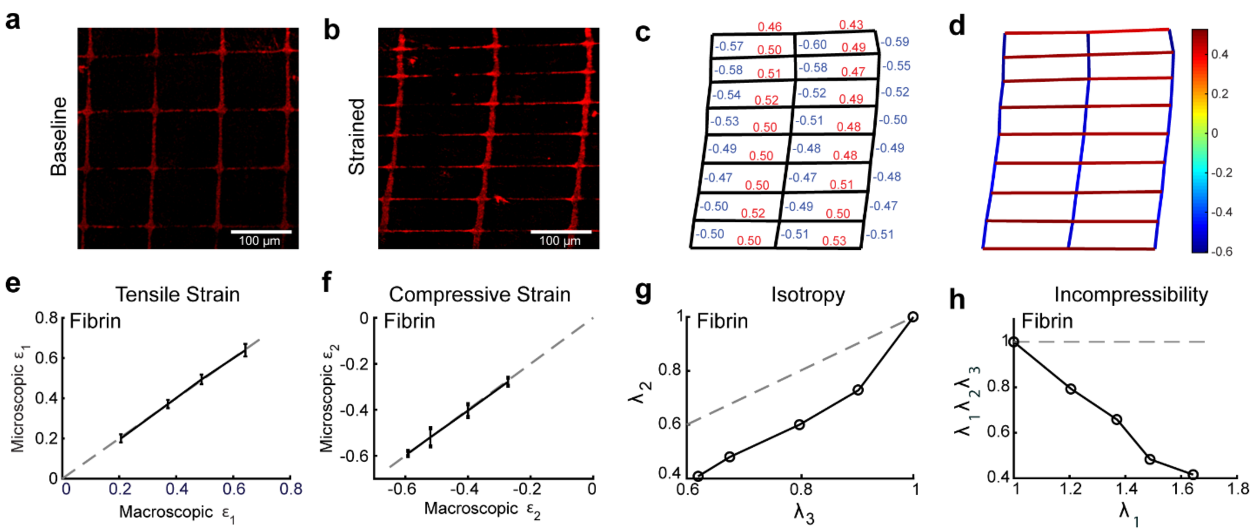

Supplementary Fig. 3 | Uniaxial tensile testing and Finite Element Analysis of the NMBS on a Fibrin test strip. a. Example wide-field fluorescence image of the pre-strain NMBS (100 µm X 100 µm X 10 µm) on a fibrin test strip. **b**. Representative fluorescence image of 50% strained NMBS following tensile testing. **c**. Image segmentation and analysis of NMBS nodes and segments following 50% uniaxial tensile (positive, red) and compressive (negative, blue) mechanical strain. **d**. A color map indicating the locations and magnitude of tensile (red) and compressive (blue) mechanical strains. **e.** The microscopic tensile strain ε_1 of the NMBS strongly correlates with the macroscopic tensile strain ε_1 of fibrin (mean ± S.D; n = 18 segments over 1 experiment). **f**. The microscopic compressive strain ε_2 of the NMBS strongly correlates with the macroscopic compressive strain ε_2 of fibrin (mean ± S.D; n = 24 segments over 1 experiment). **g.** For fibrin gels the There stretch ratios λ_2 and λ_3 were not equivalent confirming that fibrin is an anisotropic material. **h.** The product of the stretch ratios $\lambda_1\lambda_2\lambda_3$ was less than 1 during uniaxial stretching confirming that fibrin is compressible.

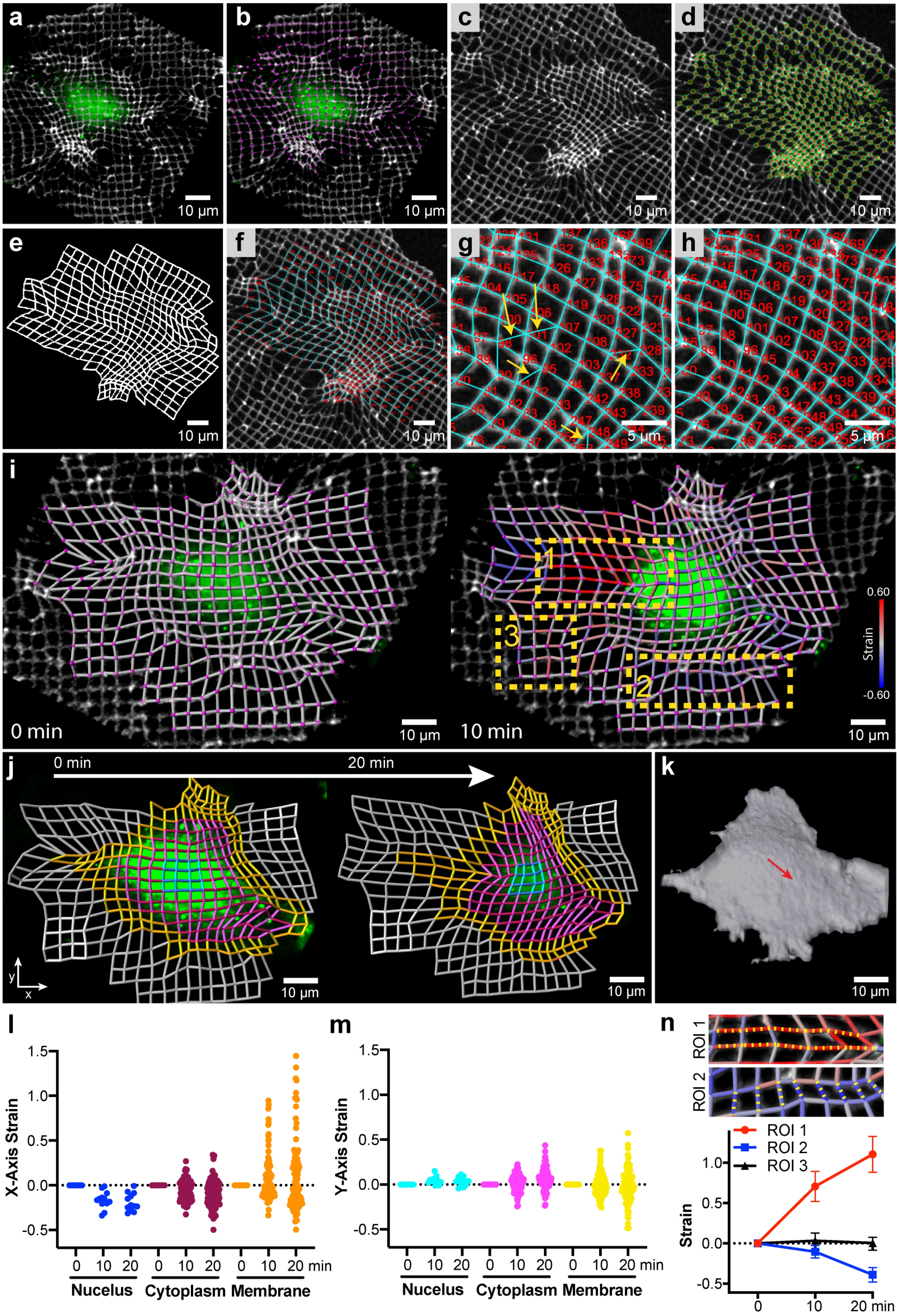
a
b
c
d
e
f
g
h
10 μm
5 μm
i
0 min
10 min
1
2
3
0.60
Strain
-0.60
j
0 min
20 min
y
x
k
l
X-Axis Strain
1.5
1.0
0.5
0.0
-0.5
0 10 20 0 10 20 0 10 20 min
Nucelus
Cytoplasm
Membrane
m
Y-Axis Strain
Nucelus
Cytoplasm
Membrane
n
ROI 1
ROI 2
ROI 3
Strain
0 10 20 min

Supplementary Fig. 4 | Custom Imaris and Matlab segmentation and tracking of NMBS images for surface strain quantification. **a.** Confocal fluorescence image of 633-FN-NMBS (2 µm X 2 µm X 2 µm, Gray) applied to HSMC incubated with 488-CellTracker (Green) and visualized in Imaris 3D visualization software. **b.** NMBS mesh node detection (Magenta) overlaid onto NMBS. **c.** Maximum intensity Z-projection of NMBS imported into Matlab from Imaris. **d.** X and Y node coordinates (Green circles) exported from Imaris to Matlab and overlaid onto NMBS maximum intensity Z-projection for pair determination. **e.** Skeletonized binary image of the NMBS mesh defined by the node coordinates. **f.** Computationally generated pair determination via pathfinding algorithm. **g.** Example errors in pair determination (yellow arrows). Numbers represent the assigned i.d. for each individual paired line segment. **h.** Manual correction of wrongly assigned pair-wise connections. **i.** Paired segments between connected nodes imported from Matlab to Imaris with a custom statistic for mapping engineering strain (baseline strain = 0, gray; tension = positive values, red; compression = negative values, blue). **j.** Manual segmentation of subcellular regions for comparative strain quantification over time in the horizontal (X-axis) and vertical (Y-axis) starting orientations for the nucleus (X = blue, Y = cyan), cytoplasm (X = red, Y = magenta), and peripheral membrane (X = orange, Y = yellow). **k.** Displacement vector (red arrow) following cell tracking showing direction of HSMC migration during imaging sequence. **l.** Quantification of all horizonal X-axis NMBS filaments show a trend of increased compression in the nucleus (more negative) and increased variability in the peripheral membrane region suggesting localized compression and tension within this region. **m.** Analysis of the vertical Y-axis strain segments revealed less overall change than seen in the horizontal segments, but increased variability over time due to smaller localized effects. **n.** Two subregions of interest were defined within the peripheral membrane area (ROI 1, tensile region; ROI 2, compressive region) showing large changes in strain "i" were extracted for quantitative strain analysis. ROI 1 located at the cells trailing edge displayed increased tensile strain while ROI 2 located at the leading edge showed increasing compressive strain as the cell migrated to the right of the image (mean ± S.D; ROI 1 n = 10, and ROI 2 n = 8 segments over 1 experiment). ROI 3, an area of the

NMBS adjacent to the cell exhibited minimal change in segmental strain over time as expected (mean ± S.D; ROI 3 n = 24 segments over 1 experiment). At both 10 and 20 min ROI 1 and ROI2 were statistically significant from time 0 min, and were significantly different at each time point compared to the adjacent ROI 3 (at 10 min, the CI = 0.4787 to 0.8692 with $p = 0.00001737$ for ROI 1 vs. ROI 3, and the CI = -0.2332 to -0.03957 with $p = 0.00678332$ for ROI 2 vs. ROI 3; at 20 min, the CI = 0.8669 to 1.332 with $p = 0.00000325$ for ROI 1 vs. ROI 3, and the CI = -0.5011 to -0.2874 with $p = 0.00000758$ for ROI 2 vs. ROI 3 by Two-way ANOVA with Tukey multiple comparisons).

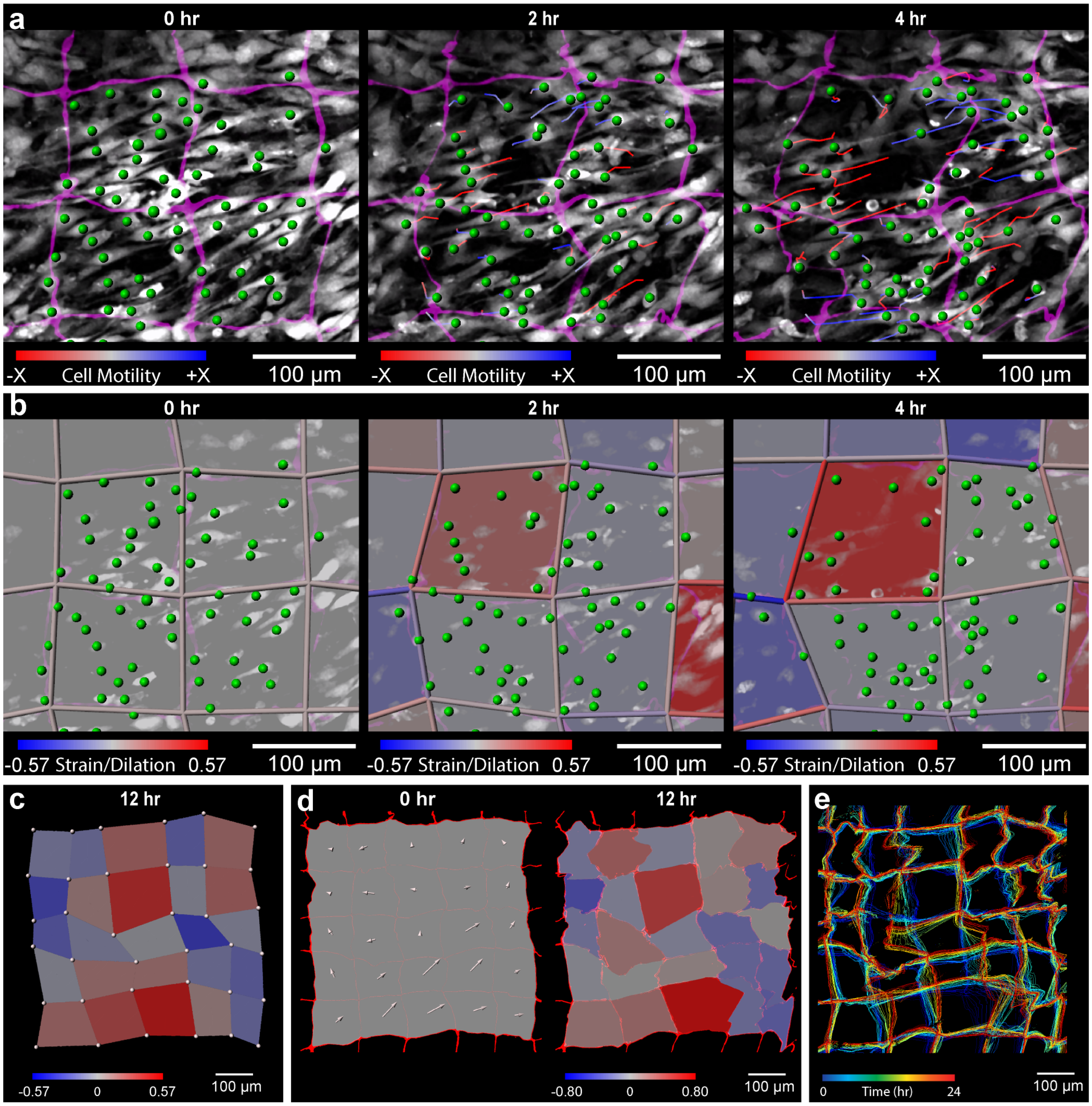

Supplementary Fig. 5 | Overall cell motility direction and morphological changes underly NMBS deformation. a. Cell motility tracking (green spots) in Imaris of C2C12 cells following transition to differentiation media within a 2 by 2 subregion of the NMBS. Dragon tails show the track displacement for a 1-hour history for each cell colored for X-axis displacement direction (Negative = Red, Positive = blue). **b.** C2C12 cell positions (green spots) over time overlaid onto the NMBS strain and dilation analysis. **c.** C2C12 area dilation analysis following 12 hours in differentiation media measured from NMBS node-segment analysis method. **d.** Regional displacement (white arrows) and area dilation analysis using a 2D maximum intensity projection of the NMBS followed by regional segmentation. Comparison of this

dilation analysis approach to the node-node linear quantification in "c" demonstrates a similar overall trend in dilation with minor differences resulting for deformations within the NMBS region between nodes **e.** Time-stacked image of the NMBS applied to C2C12 cells using the 2D maximum intensity projection showing all deformations over a 24-hour period following transition to differentiation media.

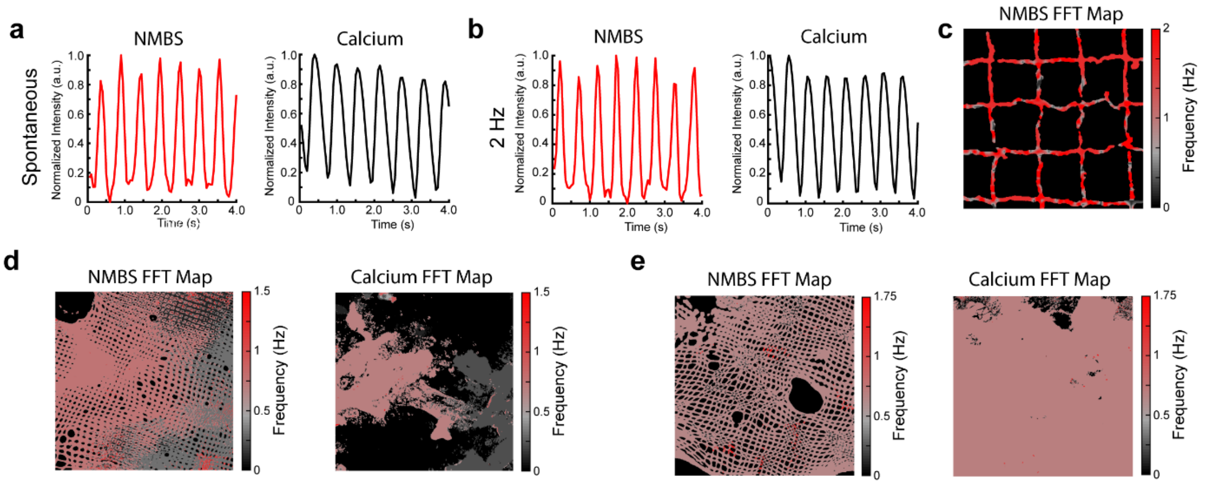

Supplementary Fig. 6 | Utilizing the NMBS to track functional cardiomyocyte performance. a. Pixel level changes in fluorescence intensity for NMBS and calcium (Fluo-4 dye) during spontaneous iPSC-derived cardiomyocyte contractions. **b.** Changes in fluorescence intensity for NMBS and calcium (Fluo-4 dye) during 2 Hz field stimulation of iPSC-derived cardiomyocytes. **c.** Fast Fourier Transform (FFT) map of predominant cardiomyocyte beat frequency obtained from NMBS (100 µm X 100 µm X 10 µm) fluorescence motion following 2 Hz field stimulation. **d.** Heterogeneous FFT map from NMBS (20 µm X 20 µm X 10 µm) and calcium (Fluo-4 dye) of spontaneously contracting iPSC-derived cardiomyocytes. **e.** Homogeneous FFT map from NMBS (20 µm X 20 µm X 10 µm) and calcium (Fluo-4 dye) of spontaneously contracting iPSC-derived cardiomyocytes.

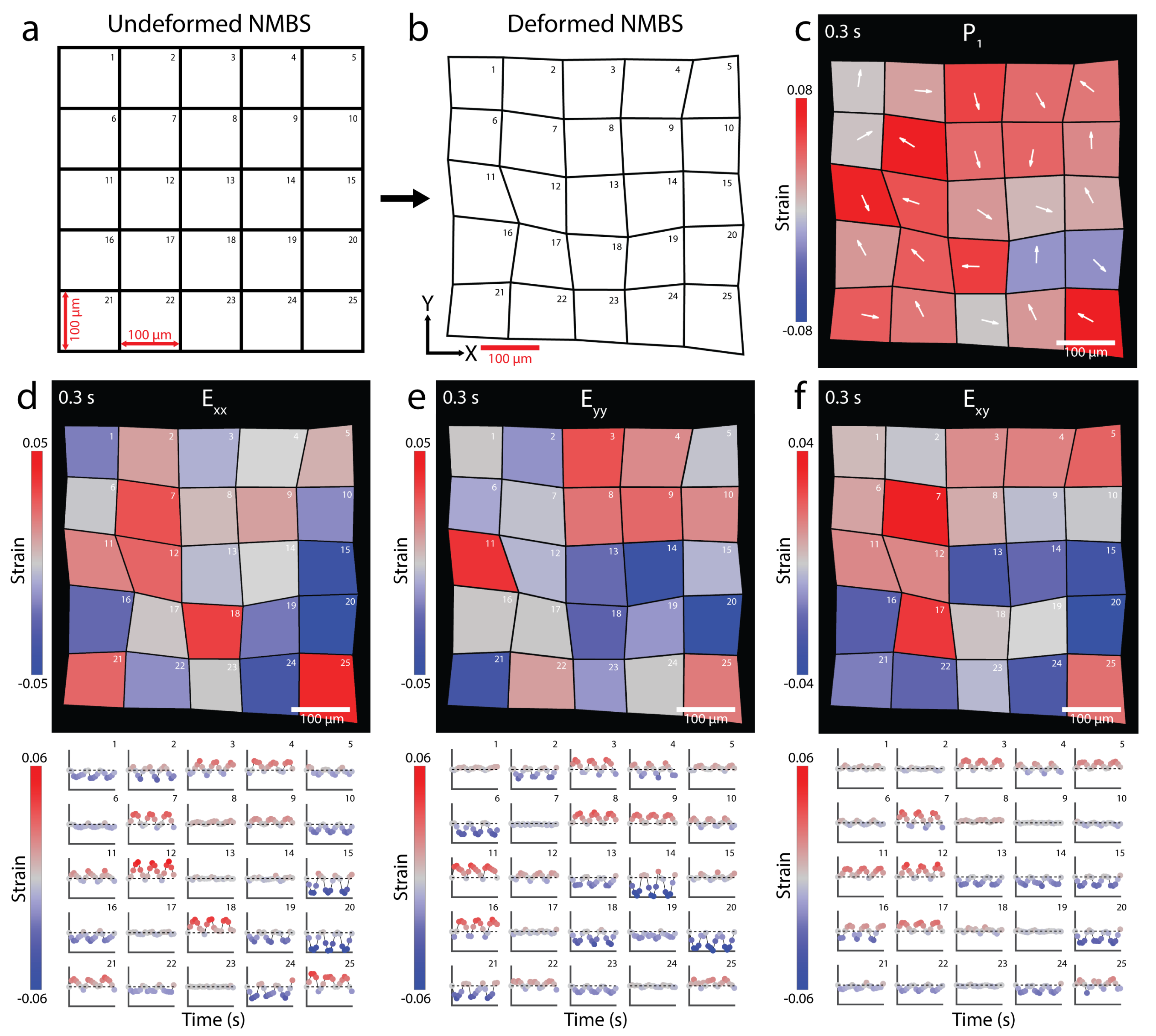

Supplementary Fig. 7 | 2D strain analysis from the NMBS on contractile cardiomyocytes. a. The undeformed NMBS state based on the 100 μm X 100 μm X 10 μm fabrication dimensions. **b.** The deformed state of the NMBS after application to the cardiomyocyte monolayer and at 0.3 s into time-lapse imaging corresponding to peak systole. **c.** Regional principle strain (tension, red; compression, blue) and direction (white arrows) for the NMBS at peak systole during a contraction cycle. **d-f.** Analysis of X-strain (E_{xx}), Y strain (E_{yy}), and the shear strain (E_{xy}) at peak systole (0.3 s, above) and over a 2 second time interval (below) defined by the NMBS meshed regions (tension, red; compression, blue).

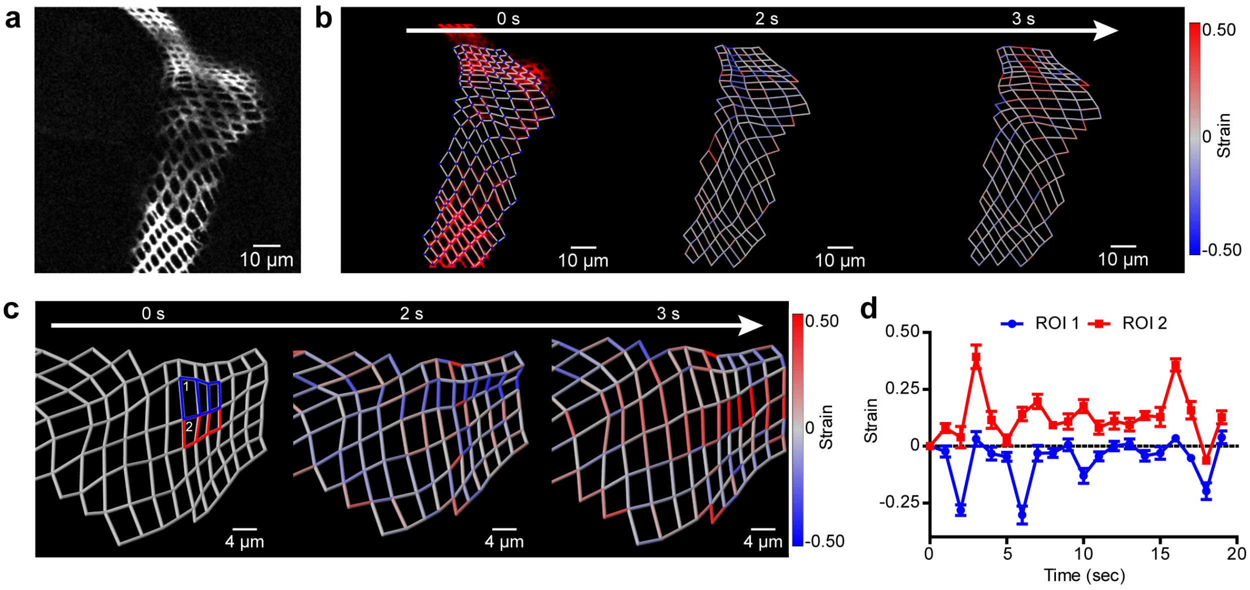

Supplementary Fig. 8 | Live strain via NMBS mapping during ovariole muscle sheath contractions. **a.** Maximum intensity Z projection of spinning disk confocal fluorescence images of a region of NMBS applied to a drosophila ovariole. **b.** Overlay of segmented and tracked NMBS (2 µm X 2 µm X 2 µm) segments and nodes onto the NMBS fluorescence image. Tension (red) and compression (blue) are regionally localized during ovariole muscle contractions. **c.** Region of interest (ROI 1 (blue), and 2 (red)) that undergoes large compressive and tensile strain over time. **d.** Quantification of ROI 1 and 2 showing oscillations in strain during ovariole contractile motion (mean ± S.D; n = 6 segments for both ROI 1 and 2 over 1 experiment).
