# Supplementary material for: Fibronectin-based nanomechanical biosensors to map 3D surface strains in live cells and tissue
Source: Nat Commun. 2020 Nov 18;11:5883. doi: 10.1038/s41467-020-19659-z (PMC7675982; doi:10.1038/s41467-020-19659-z)
Supplement: Supplementary file 15 — Description of Additional Supplementary Files [file 41467_2020_19659_MOESM15_ESM.docx]

**Description of Additional Supplementary Files**

File Name: Supplementary Movie 1

Description: NMBS Strain Mapping of HSMC During Migration

File Name: Supplementary Movie 2

Description: NMBS Strain Mapping of C2C12 Monolayer During Differentiation

File Name: Supplementary Movie 3

Description: C2C12 Region Showing Large Change in Strain

File Name: Supplementary Movie 4

Description: NMBS on Contracting Cardiomyocytes with Calcium Imaging

File Name: Supplementary Movie 5

Description: Example Region for Fast Fourier Transform Frequency Analysis

File Name: Supplementary Movie 6

Description: NMBS on Contracting Cardiomyocytes Region 2

File Name: Supplementary Movie 7

Description: NMBS on Contracting Cardiomyocytes Region 3

File Name: Supplementary Movie 8

Description: NMBS Strain Mapping of Contracting Cardiomyocytes Region 1

File Name: Supplementary Movie 9

Description: Principle strain and direction map over time for Contracting Cardiomyocytes

File Name: Supplementary Movie 10

Description: Z-stack Showing NMBS Conforming to Drosophila Ovariole Surface

File Name: Supplementary Movie 11

Description: 3D NMBS Strain Mapping of Drosophila Ovariole

File Name: Supplementary Movie 12

Description: 3D NMBS Strain Mapping of Contracting Drosophila Ovariole 1

File Name: Supplementary Movie 13

Description: 3D NMBS Strain Mapping of Contracting Drosophila Ovariole 2
