# Supplementary material for: Fibronectin-based nanomechanical biosensors to map 3D surface strains in live cells and tissue
Source: Nat Commun. 2020 Nov 18;11:5883. doi: 10.1038/s41467-020-19659-z (PMC7675982; doi:10.1038/s41467-020-19659-z)
Supplement: Supplementary file 16 — Reporting Summary [file 41467_2020_19659_MOESM16_ESM.pdf]

nature research

Corresponding author(s): Adam Feinberg

Last updated by author(s): Sep 23, 2020

Reporting Summary

Nature Research wishes to improve the reproducibility of the work that we publish. This form provides structure for consistency and transparency in reporting. For further information on Nature Research policies, see Authors & Referees and the Editorial Policy Checklist .

Statistics

For all statistical analyses, confirm that the following items are present in the figure legend, table legend, main text, or Methods section.

n/a	Confirmed	
☐	☒	The exact sample size (n) for each experimental group/condition, given as a discrete number and unit of measurement
☐	☒	A statement on whether measurements were taken from distinct samples or whether the same sample was measured repeatedly
☐	☒	The statistical test(s) used AND whether they are one- or two-sided<br>*Only common tests should be described solely by name; describe more complex techniques in the Methods section.*
☐	☒	A description of all covariates tested
☐	☒	A description of any assumptions or corrections, such as tests of normality and adjustment for multiple comparisons
☐	☒	A full description of the statistical parameters including central tendency (e.g. means) or other basic estimates (e.g. regression coefficient) AND variation (e.g. standard deviation) or associated estimates of uncertainty (e.g. confidence intervals)
☐	☒	For null hypothesis testing, the test statistic (e.g. F, t, r) with confidence intervals, effect sizes, degrees of freedom and P value noted<br>*Give P values as exact values whenever suitable.*
☒	☐	For Bayesian analysis, information on the choice of priors and Markov chain Monte Carlo settings
☒	☐	For hierarchical and complex designs, identification of the appropriate level for tests and full reporting of outcomes
☒	☐	Estimates of effect sizes (e.g. Cohen's d, Pearson's r), indicating how they were calculated

Our web collection on statistics for biologists contains articles on many of the points above.

Software and code

Policy information about availability of computer code

Data collection	For NMBS pattern design we used AutoCAD (Autodesk) 2018. To acquire atomic force microscopy images we used Asylum Research AFM Software version 14.23.153 and IGOR Pro 6.1 software (WaveMetrics) for analysis. For fluorescence imaging we used a variety of software depending on the microscope used. Widefield fluorescence imaging was performed using Nikon NIS Elements AR 4.0, and ImageJ Micromanager v1.46. Scanning confocal fluorescence imaging was acquired using Nikon NIS Elements Ar 5.11, and Zeiss Zen 2012 SP5. Spinning disk confocal fluorescence imaging was acquired using Andor IQ2.2 software. 3D CAD models were made in Fusion 360 (Autodesk) 2019 release. Finite element modeling was performed using ANSYS Mechanical 2019.
Data analysis	General image analysis and visualization was done in FIJI ImageJ software version 1.52t. All custom analysis code for NMBS strain identification, tracking, and 3D mapping was performed using Imaris 9.5.1 (Bitplane) and MATLAB (MathWorks) 2019. Each individual analysis code (XTSpotsToFilamentsWithStrain.m, XTFilamentLengthStrain.m, XTCellAreaDilation.m, BeatFrequencyFFTMapping.m, totalStrainCode.m, plotImarisDilation.m) are all version 1.0 and is available as open source at Zenodo.org (https://doi.org/10.5281/zenodo.4065743). Finite element analysis was performed and evaluated using ANSYS Mechanical 2019. All data graphing and statistical analysis was performed with GraphPad Prism 7 and Microsft Excel (v16) software. Figure preparation and adjustment of image levels was performed using Adobe Illustrator and Photoshop CS6. Editing of supplemental videos was done using Adobe Premier Pro CS6.

For manuscripts utilizing custom algorithms or software that are central to the research but not yet described in published literature, software must be made available to editors/reviewers. We strongly encourage code deposition in a community repository (e.g. GitHub). See the Nature Research guidelines for submitting code & software for further information.

Data

Policy information about availability of data

All manuscripts must include a data availability statement. This statement should provide the following information, where applicable:

- Accession codes, unique identifiers, or web links for publicly available datasets
- A list of figures that have associated raw data
- A description of any restrictions on data availability

Source data are provided with this paper. The raw image data used for strain mapping of PDMS, HSMCs, C2C12 cells, and Drosophila ovarioles in this manuscript are available as open source at Zenodo.org. (https://doi.org/10.5281/zenodo.4064033). Figures with raw data provided are: Figures 2, 3, 4, and 5, along with Supplementary Figs. 2, 6, and 8.

Field-specific reporting

Please select the one below that is the best fit for your research. If you are not sure, read the appropriate sections before making your selection.

[x] Life sciences [ ] Behavioural & social sciences [ ] Ecological, evolutionary & environmental sciences

For a reference copy of the document with all sections, see nature.com/documents/nr-reporting-summary-flat.pdf

Life sciences study design

All studies must disclose on these points even when the disclosure is negative.

Sample size	Statistical tests were chosen based on the experimental sample size, distribution, and data requirements. In all cases, the relevant reproducibility for evaluating the NMBS as a strain sensor was our ability to apply, detect, and analyze individual segments within the NMBS lattice. Therefore, our use of statistical analysis in this manuscript was not to determine a statistical difference between two experimental groups, but rather to calculate the reproducibility of measurement for the NMBS within a given experiment. For this reason, all error bars are shown as mean with standard deviation within a given experiment over time and the n values are described as the number of individual lattice segments used for quantification. For each experiment, sample sizes were checked to insure adequate data and segment numbers were acquired and analyzed to demonstrate the capabilities of the NMBS following quantitative evaluation of the data.
Data exclusions	No data was excluded in experiments that were deemed successful, and therefore was included in further analysis. Unsuccessful experiments were ones in which the NMBS did not transfer to cells or tissue, and there was therefore no data to collect. For all data sets analyzed in the manuscript, no data was selectively excluded.
Replication	Replication of study was not appropriate for this manuscript. In our case, validation of our NMBS platform across multiple type of cells, tissue, and materials was proof of replication and use of the method. Specifically, we validated our method for strain tracking across synthetic hydrogels, biological hydrogels, single cells, cell monolayers, and developing tissue demonstrating the reproducibility in NMBS application to a variety of materials and the robustness of our analysis in various experimental settings.
Randomization	Randomization was not relevant to this study. The creation of a new strain biosensor and subsequent validation did not require experimental group randomization.
Blinding	Data blinding was not relevant for this study because the analysis was unbiased and validation of a direct strain biosensor in the NMBS produced visual deformation that was computationally quantified. The process of analysis was completely automated and introduced no bias that required blinding.

Reporting for specific materials, systems and methods

We require information from authors about some types of materials, experimental systems and methods used in many studies. Here, indicate whether each material, system or method listed is relevant to your study. If you are not sure if a list item applies to your research, read the appropriate section before selecting a response.

Materials & experimental systems

n/a	Involved in the study	
x		Antibodies
	x	Eukaryotic cell lines
x		Palaeontology
	x	Animals and other organisms
x		Human research participants
x		Clinical data

Methods

n/a	Involved in the study	
x		ChIP-seq
x		Flow cytometry
x		MRI-based neuroimaging

Eukaryotic cell lines

Policy information about cell lines

Cell line source(s)	For our investigations of cellular strain using the NMBS we used human skeletal muscle cells (HSMC, Cook MyoSite SK-1111), C2C12 mouse myoblast cells (CRL-1772, ATCC), and HES3 human embryonic stem cell (hESC)-derived cardiomyocytes were received from ESI and WiCell (ES03).
Authentication	HSMC (Cook MyoSite SK-1111) were tested to be ≥ 70% cells positive for desmin and C2C12 (ATCC® CRL-1772) were tested for myogenesis following growth to confluency and formation of myotubes after 14 days in culture. Both lines were deemed authentic for contractile muscle phenotypes as evident by myotube formation experiments. In our experiments, the HES3 hESC were differentiated and evaluated for contractility and calcium handling for phenotype authentication. No genomic authentication was performed.
Mycoplasma contamination	All cells used in this study tested negative for mycoplasma contamination. To identify cytoplasm contamination we routinely perform fluorescence imaging to check for the presence of mycoplasma with DNA staining. Additionally, if suspected, we will quarantine that cell line in question and confirm with mycoplasma PCR assay kit; however, this was not needed. We rarely have issues with mycoplasma contamination in the lab, and order new fresh authenticated C2C12 and HSMCs every 6 months when needed from ATCC and Cook Myocyte.
Commonly misidentified lines (See ICLAC register)	No commonly misidentified lines were used.

Animals and other organisms

Policy information about studies involving animals; ARRIVE guidelines recommended for reporting animal research

Laboratory animals	The Drosophila Fly stocks used in this study were Moesin-GFP/TM6,Tb59,60 and P{His2Av-mRFP1}III.1 (Blooming Drosophila Stock Center #23650). A fly stock containing both Moesin-GFP and H2Av-RFP was generated by crossing the aforementioned parental fly lines (P{His2Av-mRFP1}III.1, Moesin-GFP/TM6,Tb). For our experiments ovaries were dissected from 1 week old female flies, and the ovarioles were collected for application of the NMBS.
Wild animals	This study did not involve wild animals.
Field-collected samples	This study did not involve field collected samples.
Ethics oversight	Animal approval is not need for the invertebrate Drosophila model organism used in this study.

Note that full information on the approval of the study protocol must also be provided in the manuscript.
